# Creating Chemiluminescence Signature Arrays Coupled with Machine Learning for Alzheimer’s Disease Serum Diagnosis

**DOI:** 10.34133/research.0653

**Published:** 2025-05-12

**Authors:** Biyue Zhu, Yanbo Li, Shi Kuang, Huizhe Wang, Astra Yu, Jing Zhang, Jun Yang, Johnson Wang, Shiqian Shen, Xuan Zhai, Jiajun Xie, Chongzhao Ran

**Affiliations:** ^1^ Athinoula A. Martinos Center for Biomedical Imaging, Department of Radiology, Massachusetts General Hospital/Harvard Medical School, Boston, MA 02129, USA.; ^2^ Department of Pharmacy, National Clinical Research Center for Child Health and Disorders, Ministry of Education Key Laboratory of Child Development and Disorders, China International Science and Technology Cooperation Base of Child Development and Critical Disorders, Chongqing Key Laboratory of Child Neurodevelopment and Cognitive Disorders, Children’s Hospital of Chongqing Medical University, Chongqing 400014, China.; ^3^ 99 Vista Montana, San Jose, CA 95134, USA.; ^4^ Department of Anesthesia, MGH Center for Translational Pain Research, Critical Care and Pain Medicine, Massachusetts General Hospital, Harvard Medical School, Boston, MA, USA.

## Abstract

Although omics and multi-omics approaches are the most used methods to create signature arrays for liquid biopsy, the high cost of omics technologies still largely limits their wide applications for point-of-care. Inspired by the bat echolocation mechanism, we propose an “echoes” approach for creating chemiluminescence signatures via screening of a compound library, and serum samples of Alzheimer’s disease (AD) were used for our proof-of-concept study. We first demonstrated the discrepancy in physicochemical properties between AD and healthy control serums. On this basis, we developed a simple, cost-effective, and versatile platform termed UNICODE (UNiversal Interaction of Chemiluminescence echOes for Disease Evaluation). The UNICODE platform consists of a “bat” probe, which generates different chemiluminescence intensities upon interacting with various substrates, and a panel/array of “flag” molecules that are selected from library screening. The UNICODE array could enable the reflecting/“echoing” of the signatures of various serum components and intact physicochemical interactions between serum substrates. In this study, we screened a library of over 1,000 small molecules and identified 12 “flag” molecules (top 12) that optimally depict the differences between AD and healthy control serums. Finally, we employed the top 12 array to conduct tests on serum samples and utilized machine learning methods to optimize detection performance. We successfully distinguished AD serums, achieving the highest area under the curve of 90.24% with the random forest method. Our strategy could provide new insights into biofluid abnormality and prototype tools for developing liquid biopsy diagnoses for AD and other diseases.

## Introduction

Blood serum, one of the most accessible biofluids, has been extensively explored for its diagnostic value due to its vital role in supporting biological functions and physiological processes [1–5]. Recently, serum diagnostic technologies have been rapidly developed, including liquid biopsy [1–5]. While numerous advanced technologies have demonstrated promising detection capabilities, their high costs hinder broad applications in large-scale screening. To date, there is a growing demand for simple, versatile, and cost-effective detection methods to enhance the primary screening process and support subsequent evaluations. However, limited tools could be easily applied to improve screening of diseases, such as Alzheimer’s disease (AD) [6].

Liquid biopsy is shifting from traditional single-readout-per-sample approaches to signature-based readouts, with omics and multi-omics technologies most employed to generate comprehensive signatures. Omics approaches focus on identifying a range of specific molecules (proteins, lipids, etc.) to create a panel or array that mirrors the signature of a disease [7–10], and notable advancements in using omics for liquid biopsy have been reported [11–13]. However, the high costs associated with omics technologies have largely limited their accessibility in low-cost disease screening and point-of-care settings. Exploring new methods that enable simple profiling of disease signatures holds great potential for complementing existing primary screening strategies [6].

Current diagnostic methods, including omics technologies, are mainly based on detecting the abundance of biomolecules and identifying disease-specific biomarkers. However, the abnormality of physicochemical interactions in serum has been largely overlooked. In fact, molecular interaction between proteins, lipids, carbohydrates, and small molecules is a universal phenomenon in biological systems [14–16]. The universal interactions between molecules vary in strength and specificity. Most of these interactions fall into competitive and cooperative modes, including synergic or allosteric mechanisms [14–16]. These interactions are strongly related to biomolecules’ physicochemical properties (hydrophobicity, conformation, etc.) and could be changed under disease conditions [14–16]. Hypothetically, beyond the abundance signature, the readout of each physicochemical interaction could also provide signatures to reflect the status of the serum. However, conventional serum diagnosis approaches, including omics, are limited to generating signatures of physicochemical abnormality [17]. Importantly, omics approaches have limited capacity for generating physicochemical signatures because the process/isolation of proteins, lipids, etc., could destroy physicochemical interactions. In addition, current physicochemical characterization methods for serum are tedious and have low throughput. Hence, physicochemical abnormality has been largely overlooked and rarely explored for its diagnostic value [11–13].

**Fig. 1.**
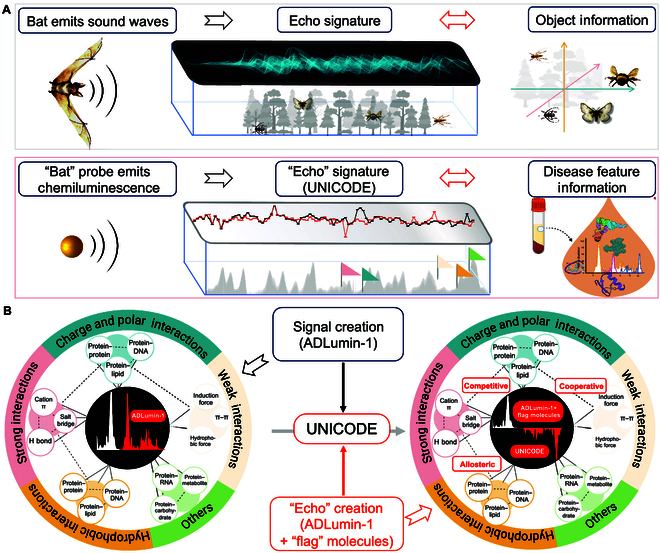
Diagram of the principle of UNICODE (UNiversal Interaction of Chemiluminescence echOes for Disease Evaluation). (A) Bat echolocation is a smart detecting system in nature. When passing through an unknown environment, a bat emits a sound wave that bounces off an object, returning an echo that provides information about the distance and size of the object. Inspired by this phenomenon, we hypothesized that a “bat” probe emits a signal that bounces off a biomolecule, returning an echo for recognizing surrounding changes could be used to depict serum signatures. (B) ADLumin-1 is a “bat” probe that emits distinct chemiluminescence signals based on the surrounding physicochemical environment. We hypothesize that introducing “flag” molecules could perturbate this interaction by competitive, cooperative, or allosteric mechanisms and thus enable serum signature profiling. By screening the best “flag” molecule for a specific disease, it is possible to profile the disease features in the biofluids and develop a diagnosis.

Bat echolocation is a smart detecting system in nature [18]. As a bat navigates through an unfamiliar environment, it emits sound waves that interact with surrounding objects, each of which alters the properties of the sound. The modified waves then echo back to the bat, providing information about the characteristics of the objects it encountered. Since different objects produce distinct echo patterns, the bat can use these features to generate echo signatures, allowing it to determine the distance and size of the objects in its flight path [18,19]. Inspired by this phenomenon, we hypothesized that a “bat” probe could emit signals, like chemiluminescence, to interact with biomolecules in the environment. These interactions would modify the emitted signals, and the altered signals could then be used to generate unique signatures that reflect changes in the physicochemical environment of serum components (Fig. 1A). To achieve this goal, the “bat” probe should meet the following requirements: (a) it generates signals spontaneously with a high signal-to-noise ratio (SNR); (b) the signal change is related to the physicochemical environment of biomolecules; and (c) its signals can be altered upon interacting with biomolecules, and the substantially altered signals can be assembled into a unique signature to reflect the interactions.

Chemiluminescence imaging is a powerful tool for sensing biomolecular targets [20–23]. Due to their self-illuminating capability, high sensitivity, and low cost, chemiluminescence probes have attracted increasing interest in exploring their medical diagnostic potential [20–23]. However, activating most chemiluminescence probes requires enzymes or reactive oxygen species (ROS), such as hydrogen peroxide, which hampers their applications for sensing the intrinsic physicochemical properties of serum [20–23]. Recently, we have reported a chemiluminescence probe (ADLumin-1) [24]. Different from other catalyst-requiring probes, ADLumin-1 could spontaneously emit chemiluminescence signals with a high SNR when interacting with various proteins, especially beta-sheet-enriched structures [24]. With the molecular rotor scaffold, ADLumin-1 flexibly rotates in a solution and emits signals based on the degree of the restriction of the rotation of chemical bonds, which is strongly related to the conformational structure and physicochemical environment of the targets. This spontaneous characteristic of ADLumin-1 is similar to that of a bat that emits sound waves. In this regard, we hypothesized that ADLumin-1 could be considered a “bat” probe to report the physicochemical properties of serum components.

Traditionally, a single chemiluminescence probe with one sample yields only one averaged (summed) readout of the diverse interactions in the serum; however, such an averaged readout totally masks the underlying complexity of these physicochemical interactions. To “recover” the diversity, signature readouts are needed. To establish the signature, we speculated that (a) the universal interactions of ADLumin-1 with disease-specific (signature) proteins could be perturbated if there is a competitive, collaborative, or allosteric binder for the same proteins, and this perturbation could be detected via the chemiluminescence of ADLumin-1, and (b) by screening a compound/binder library, we could identify a panel of binders that substantially alter these chemiluminescence signals. This panel would allow us to create a signature for the “bat” probe, ADLumin-1, which would “echo” the diversity of physicochemical interactions in a serum sample. In this analogy, much like “flag” objects in a bat’s echo path, we consider these selected molecules as “flag” molecules. Therefore, the combination of a “bat” probe and “flag” molecules generates a series of signature “echoes” that reveal the serum’s unique profile. Based on this principle, we developed an assay termed UNICODE (UNiversal Interaction of Chemiluminescence “echOes” for Disease Evaluation) to evaluate the physicochemical abnormality of serum (Fig. 1B).

In this report, leveraging the high sensitivity of ADLumin-1 as the “bat” probe and the versatility of “flag” molecule panels, we created distinct signatures for various serum components and subtle physicochemical environments. Specifically, we used AD, the most devasting neurodegenerative disease that faces high demand for primary screening [6–10], as an example for the proof-of-concept study. We developed an AD-specific UNICODE array with 12 “flag” molecules that optimally depict the differences between AD and healthy control (HC) serums. In addition, we used machine learning (ML) methods to analyze the UNICODE signature and developed a primary screening model for AD. We believe that the UNICODE assay has the potential to be a universal signature profiling platform for AD and other chronic diseases.

## Results

### Physicochemical differences between AD and HC serum proteins

**Fig. 2.**
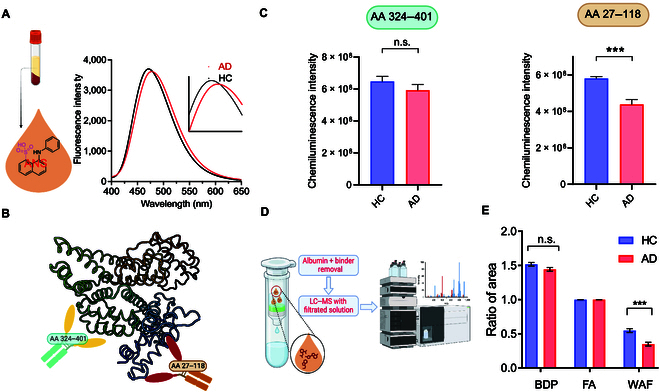
(A) Representative fluorescence emission spectra of 8-anilino-1-naphthalenesulfonic acid (ANS) with Alzheimer’s disease (AD) and healthy control (HC) serums (*n* = 3). Inset: zoom-in of the range of 450 to 500 nm. (B) Diagram of the structures of albumin and the epitopes (amino acids 27 to 118 and amino acids 324 to 401) that bind to the corresponding antibodies. (C) Quantitative analysis of epitope mapping for serum samples from AD (*n* = 3) and HC (*n* = 3) groups. Each serum sample was tested in 3 replicates. By dot-blotting with albumin antibodies against the amino acids 27 to 118 and amino acids 324 to 401 epitopes, we found conformational difference in amino acids 27 to 118 between 2 groups. (D) Diagram of the process for characterizing albumin–compound interaction with a removal column and liquid chromatography–mass spectrometry (LC–MS). (E) The quantification of LC–MS peak areas of benzodiazepine (BDP), folic acid (FA), and warfarin (WAF) in elutes after the albumin removal process in (E). The results indicate the molecular interaction difference in the WAF binding domain between AD (*n* = 3) and HC (*n* = 3). Each serum sample was tested in 3 replicates. n.s., not significant. ***P < 0.001. AA, amino acids.

Protein is the major component in serum, and its physicochemical properties are pivotal for functional capacity. Hydrophobicity is a key physicochemical property for protein conformation [25–27]. First, we investigated the accessible hydrophobicity of AD patients and compared it to that of HC serums. The fluorescence dye 8-anilino-1-naphthalenesulfonic acid (ANS) is a well-validated probe for reporting the surface hydrophobicity of proteins, and this is because the sulfonic acid moiety can be exposed to the surrounding water, while the aniline/naphthalene moiety can bind to the hydrophobic surface [28]. Thus, we used ANS to investigate the surface hydrophobicity of serum proteins. We tested serum samples from AD and HC with ANS and found that there are distinguishable differences between the groups (Fig. 2A). AD serums showed longer emissions (7.0-nm maxima) and lower intensities compared to the HC serums (Fig. 2A and Fig. S1a). We also mixed ANS with serums from multiple sclerosis (MS) and diabetes (DB) patients. As shown in Fig. S1b and c, these serums also showed decreased ANS intensity compared to HC but no apparent wavelength shift, suggesting that both similarities and differences in physicochemical abnormalities could exist in different diseases. These results suggested that the total surface hydrophobicity of AD serum was indeed different from that of HC serum, and the AD serum had less accessible surface hydrophobicity.

As protein surface hydrophobicity is closely related to its conformation, we tried to use circular dichroism (CD) spectroscopy to identify the secondary structure change of serum proteins. However, the serum components are too complicated for the CD instrument to give reliable spectra (data not shown). Thus, we selected albumin, the most abundant protein in serum, for further conformational studies [29,30]. Epitope mapping, which utilized the highly selective binding of antibodies to different epitopes, was used to explore the conformational change of different sites of albumin [31–33]. We first normalized the albumin concentrations to the same level with bromocresol green (BCG) assay and used 2 antibodies to investigate different epitopes (amino acids 27 to 118 and 324 to 401) of AD and HC serum albumin. Surprisingly, we found that AD serum albumin showed lower signals for the amino acids 27 to 118 region (Fig. 2B and C) compared to HC serum albumin, suggesting the conformational difference of this albumin site in AD condition.

As albumin is a general carrier for many endogenous and exogenous biomolecules [29,30], the conformational change of the albumin site may cause molecular interaction discrepancy. To prove this, a cocktail chromatography method was used for the test (Fig. 2D). Briefly, the serum albumin was captured by a spin column that contains albumin-binding resin, followed by adding a solution of small molecules into the column and letting the solution interact with the captured albumin. The unbound small molecules flowed through the column, and the elutes were analyzed by liquid chromatography–mass spectrometry (LC–MS). To exclude the artifact caused by the resin or column, we used a cocktail of both hydrophilic (folic acid [FA]) and hydrophobic molecules (warfarin [WAF] and benzodiazepine [BDP]) for each test. FA is a water-soluble molecule that can interact with albumin. WAF was proved to bind primarily at the Sudlow I/IIA subdomain of albumin [31,34]. BDP has been proved to primarily bind to the Sudlow II/IIIA subdomain of albumin [34,35]. Interestingly, apparent lower concentrations of WAF were shown in the AD group compared to those in the HC group, while FA and BDP showed no significant difference between the groups (Fig. 2E). The results indicate the molecular interaction discrepancy of the WAF binding domain.

Taken together, the results from the above methods suggest that there are differences in accessible hydrophobicity, albumin conformation, and molecular interaction between AD and HC.

### Depicting the physicochemical abnormality of serum proteins with a “bat” probe

The above results indicate the diagnostic potential of detecting serum physicochemical differences. However, current methods are not suitable for characterizing the subtle physicochemical changes of sophisticated serum components due to low throughput, limited sensitivity, and tediousness. In addition, these methods typically yield a single, averaged (or summed) readout, which masks the complexity of the underlying physicochemical interactions [36–38].

Inspired by the bat echolocation phenomenon, we hypothesized that a “bat” probe that emits signals spontaneously could be used to depict serum signatures (Fig. 1A). Recently, we have reported a chemiluminescence probe (ADLumin-1). Similar to bats that emit sound waves, ADLumin-1 could spontaneously emit chemiluminescence signals with a high SNR when interacting with various proteins, especially beta-sheet-enriched structures [24]. With a molecular rotor scaffold, ADLumin-1 flexibly rotates in solutions and emits signals based on the degree of the restriction of the rotation of chemical bonds, which is strongly related to the conformational structure and physicochemical environment of the targets. In this regard, we hypothesized that ADLumin-1 could be used as a “bat” probe to report the physicochemical properties of serum components (Fig. 1B).

**Fig. 3.**
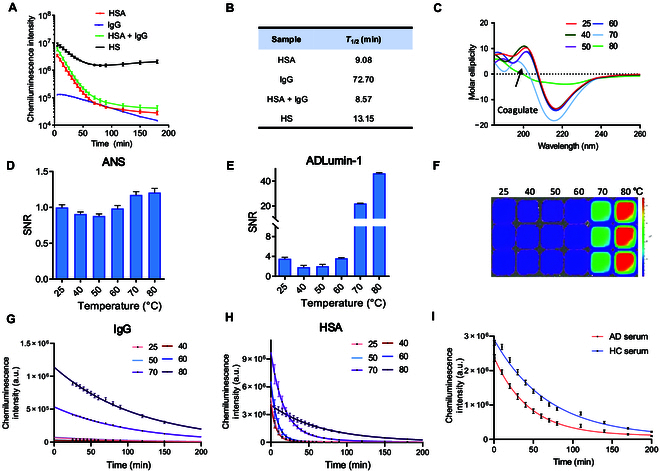
(A and B) Time course of chemiluminescence decay (A) and calculated half-lifetime (B) of a “bat” probe with 606 mM human serum albumin (HSA), 67 mM immunoglobulin G (IgG), a mixture of HSA and IgG (606 mM HSA+ 67 mM IgG), and healthy human serum (HS). The “bat” probe showed distinct chemiluminescence signal decay profiles for different samples. (C and D) Circular dichroism (CD) spectroscopy (C) and ANS assay (D) of 5 μM heated IgG proteins. After heating for 5 min at 25 to 80 °C, IgG showed conformation (C) and hydrophobicity (D) perturbation at 25 to 60 °C and destruction at 70 to 80 °C. (E to G) Enhanced chemiluminescence intensity ((E) signal-to-noise ratio [SNR]; (F) representative images) and changed signal decay profile (G) of the “bat” probe when interacting with heated IgG proteins. The data indicate its capability to detect beta-sheet perturbation and destruction. (H) Changed signal decay profile of the “bat” probe when interacting with heated albumin proteins at 25 to 80 °C. The data indicate its capability to detect alpha-helix perturbation and destruction. (I) Chemiluminescence decay profile of the “bat” probe in AD and HC serums (*n* = 14 for AD, *n* = 16 for HC).

First, we investigated whether ADLumin-1 could spontaneously emit chemiluminescence signals with serum components. Human serum albumin and immunoglobulin G (IgG) are the most representative alpha-helix and beta-sheet structures, as well as the major proteins in the serum [39,40]. Thus, albumin and IgG were selected for the test. In addition, we also prepared a mixture of 606 mM albumin and 67 mM IgG, to mimic serum conditions, and also used healthy human serum to explore their chemiluminescence signal upon ADLumin-1’s addition. The signal decay behavior showed distinct differences, and the decay half-lifetimes for albumin, IgG, albumin + IgG, and human serum are 9.08, 72.70, 8.57, and 13.15 min, respectively (Fig. 3A and B). The slow decay in the presence of IgG indicates that ADLumin-1 forms a strong and specific bond with the hydrophobic tunnel within the β-sheet-rich structure, whereas its interaction with albumin appears to be transient and nonspecific. Notably, ADLumin-1 generated various metrics of chemiluminescence signal with different samples, including the highest signal, decay profile, and plateau, and these signal features could facilitate profiling serum signatures.

Next, we investigated whether the “bat” probe could sense the physicochemical environmental changes of the serum proteins. As heating was reported to alter surface hydrophobicity and secondary structure conformation [39,40], we first prepared 5 μM heated IgG samples and mixed them with ADLumin-1 after cooling the samples to room temperature. After heating for 5 min from 25 to 80 °C, IgG samples were tested by CD spectroscopy, transmission electron microscopy (TEM), and hydrophobicity dye ANS to characterize the conformational and hydrophobicity change. CD spectra and ANS indicated conformation and hydrophobicity perturbation at 25 to 60 °C, and destruction at 70 to 80 °C (Fig. 3C and D), which were further confirmed TEM images (Fig. S2). In accordance with the CD, ANS, and TEM results, the highest signal of ADLumin-1 showed signal perturbation at 25 to 60 °C and drastic signal change at 70 to 80 °C (Fig. 3E to G), and the SNR can reach up to 38.34 ± 0.63-fold higher compared to ANS. Notably, the “bat” probe showed a distinct signal decay profile during the change of the IgG structure (Fig. 3G). We also prepared heated albumin samples and tested them in the same condition. Similarly, the “bat” probe is sensitive to albumin structure perturbation and destruction, evidenced by its enhanced signal and distinct decay profile with heated albumin (Fig. 3H). These results indicate that ADLumin-1 could sense the secondary structure alteration and reflect this change in its chemiluminescence signal and signal decay profile.

As pH, ROS, and viscosity also possibly contribute to serum features, we tested the chemiluminescence signal of ADLumin-1 under various conditions of pH, ROS, and viscosity, both with and without healthy human serum. Under pH conditions ranging from 5.5 to 8, ADLumin-1 exhibited an increased chemiluminescence signal and distinct decay profile as the pH increased (Fig. S3a). Notably, this pH-sensing ability was retained in the presence of serum (Fig. S3b). When viscosity was adjusted by varying the concentration of glycine/phosphate-buffered saline (PBS) (0% to 80% v/v), ADLumin-1 showed a higher chemiluminescence signal as viscosity increased (Fig. S3c), consistent with the known behavior of molecular rotor scaffolds that generate a turn-on phenomenon under higher viscosity [41]. In the presence of serum, the chemiluminescence signal of ADLumin-1 decreased as viscosity increased, suggesting complicated underlying mechanisms that may include biomolecular interaction and viscosity change (Fig. S3d). Furthermore, hydrogen peroxide was selected as a representative ROS to evaluate its effect on ADLumin-1’s chemiluminescence. ADLumin-1 exhibited minimal signal changes in both the presence and the absence of serum, demonstrating its stability when exposed to hydrogen peroxide (Fig. S3e and f). These results demonstrate that ADLumin-1 is sensitive to pH and viscosity. Additionally, we tested the chemiluminescence of ADLumin-1 at temperatures ranging from 25 to 80 °C. Surprisingly, the signal increased at 50 °C and decreased at 80 °C. This could be due to the enhanced molecular motion of ADLumin-1 at 50 °C, while at 80 °C, an excessively rapid signal decay may have occurred (Fig. S3g).

Inspired by the above result, we directly mixed ADLumin-1 with HC or AD serum and characterized the signal decay profile. Interestingly, we observed higher intensities and longer signal decay half-lifetime from HC serum, compared to those of AD serum (Fig. 3I and Fig. S3h and i). This indicates less accessible hydrophobic interactions, which is in accordance with the results from the ANS tests in Fig. 2A and Fig. S1.

Taken together, ADLumin-1 can serve as a “bat” probe to reflect physicochemical abnormality by its chemiluminescence signal and provide general signatures of the signal decay profile for each sample. However, further diagnosis applications require the “bat” probe to sense the subtle features of complicated serum components. Thus, an additional method should be developed to assist signature creation for the “bat” probe.

### Chemiluminescence signatures created by ADLumin-1 and “flag” molecules based on universal biomolecular interactions

Given that a specific disease is associated with unique features, the interaction between the “bat” probe and disease-specific (signature) proteins could identify the physicochemical features. However, for many diseases, the signature proteins are unknown, and it is very challenging to extract or purify them from blood samples [1,3]. Instead of characterizing each component separately, profiling the physicochemical signature of serum would be more versatile, simple, and time-saving. To overcome these barriers, we hypothesized that the universal interactions of ADLumin-1 with disease-specific (signature) proteins could be perturbated if there is a competitive, collaborative, or allosteric binder for the same proteins, and this perturbation could be detected via the chemiluminescence “echoes” of ADLumin-1. Thus, we propose to screen a compound library to identify the binders (“flag” molecule) and establish an array that can create chemiluminescence signatures to reflect the disease signatures, which we termed as a UNICODE array.

**Fig. 4.**
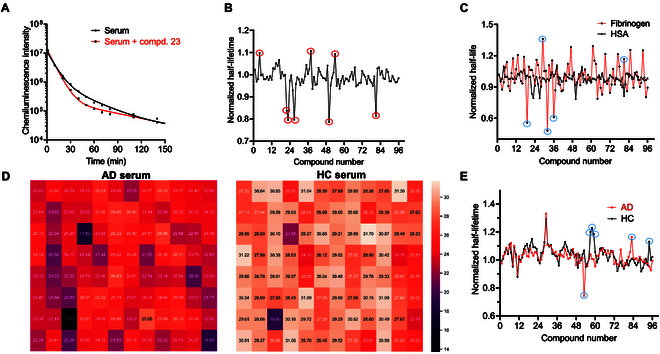
(A) Time course of the chemiluminescence decay of the “bat” probe in albumin solutions with (red line) or without (black line) a competitor compound. The addition of a competitive compound can alter the chemiluminescence intensity and decay profile. (B) Representative plot of the normalized half-lifetime of the “bat” probe/albumin against each compound; several pronounced signatures (over 3 × SD) can be observed (red circle). (C) Representative UNICODE signature mapping of the normalized half-lifetimes of human serum albumin (black) and fibrinogen (red). The top 5 flag molecules to differentiate albumin and fibrinogen are highlighted in blue circles. (D) Representative heat map of the half-lifetime of the “bat” probe/AD serum against each compound. (E) Representative UNICODE signature mapping of the normalized half-lifetimes of AD and HC serums. The top 5 flag molecules to differentiate AD and HC serums are highlighted in blue circles.

To prove this concept, we first selected albumin as an example and screened the “flag” molecules with a 96-well plate (Table S1). We mixed albumin (15.0 μM) with different compounds (2.0 μM), followed by addition of ADLumin-1 (2.5 μM), and immediately detected the chemiluminescence signal from 0 to 180 min. For imaging analysis, we used the half-lifetime (*T*_1/2_) without the compound as the baseline for normalization. As shown in Fig. 4A and B, we found that several compounds could cause apparent changes in the signal decay profile, which is over 3 × standard deviation (SD), including no. 4 (aristolochic acid), 22 (vitamin E), 23 (clofibrate), 27 (vitamin K1), 38 (dexibuprofen), 50 (idebenone), 54 (diclofenac diethylamine), and 81 (dapoxetine), which is consistent with literature reports that proved their substantial binding to albumin [42,43]. More importantly, the patterns in Fig. 4B could be considered the chemiluminescence signature for albumin, and the substantial changes caused by the “flag” molecules were the pronounced features of the signatures. We conducted representative compound screening experiments in triplicate and confirmed that these signatures exist stably (Fig. S4a). Furthermore, we investigated the effect of different concentrations of ADLumin-1 for “flag” molecule screening. By screening with representative “flag” or “non-flag” molecules for albumin, we found that concentrations of 12.5 to 50 μM of ADLumin-1 showed negligible impact on “flag” molecule selection (Fig. S4b).

To further validate the chemiluminescence “echoes” as a signature approach, we used the same compound library to perform tests with other serum components, including fibrinogen, glycoprotein, hemoglobin, and transferrin. As expected, those components showed distinct signatures from albumin and each component has unique “echo” signatures to reflect its features (Fig. S4c to f). By mapping the signatures, we successfully identified the top 5 “flag” molecules for differentiating albumin and fibrinogen (Fig. 4C).

Based on these results, we used the same 96-well array to create a UNICODE for AD and HC serums. Indeed, we found apparent signatures of chemiluminescence half-lifetime and identified several “flag” molecules (Fig. 4D and E). Interestingly, several similarities could be identified between AD and HC serums. For example, the no. 53 compound showed a lower lifetime ratio, while no. 29 indicated a high ratio for both groups, suggesting that this compound has a similar binding environment in both serum samples. Importantly, we also found specific lifetime signatures of AD serum when mapping with the signatures of HC serum (Fig. 4E). This suggests that we may differentiate AD and HC serums by screening a compound library and identifying the best “flag” molecules for AD.

Collectively, our results indicate that the combination of the “bat” probe ADLumin-1 and a panel of “flag” molecules could create chemiluminescence “echo” signatures (UNICODE) to reflect the unique features of specific serum components, AD and HC serums. In addition, UNICODE may have the potential for AD serum diagnosis.

### “Flag” molecule screening and UNICODE array with ML for diagnosis

**Fig. 5.**
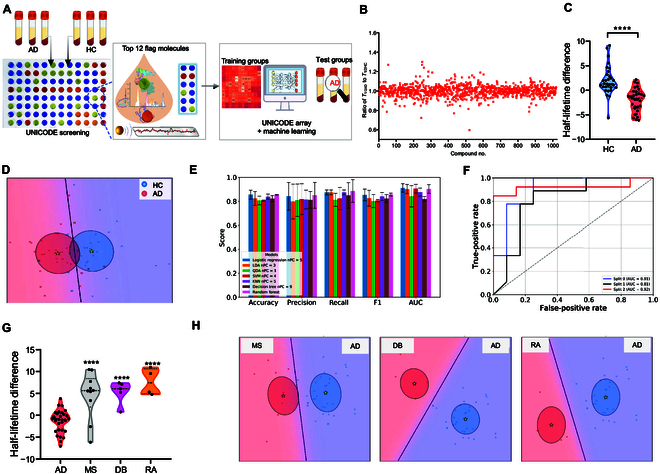
(A) Diagram of AD-specific UNICODE assay workflow. (B) A plot of the normalized half-lifetime ratio of the “bat” probe with AD serums (*n* = 3) to it with HC serums (*n* = 3) against 1,024 compounds; 12 “flag” molecules were identified as the UNICODE array for AD serum diagnosis with machine learning. (C) A representative lifetime analysis of a “flag” molecule (clofazimine) with AD serum and HC serum. (D) Classification of AD and HC cohorts with the linear discriminant analysis (LDA) method. (E) The comprehensive metric results of machine learning of the UNICODE data for AD serum (*n* = 31) and HC serum (*n* = 37). Random forest showed the best performance among all methods. Each bar is the average data of 3 independent experiments for each method. (F) Receiver operating characteristic (ROC) curve of the random forest model. (G and H) Representative half-lifetime differences analysis of a “flag” molecule (clofazimine) (G) and LDA-based classification (H) of AD serum (*n* = 31) and serums from other diseases: multiple sclerosis (MS, *n* = 10), diabetes (DB, *n* = 5), and rheumatoid arthritis (RA, *n* = 4). ****P < 0.0001. nPC, number of principal components; QDA, quadratic discriminant analysis; SVM, support vector machine; KNN, *K*-nearest neighbor; AUC, area under the curve.

Inspired by the above data, we proposed an AD-specific UNICODE workflow by screening a larger library, discovered the top “flag” molecules array for AD, and combined it with ML methods for AD primary screening (Fig. 5A). First, we screened 1,024 small molecules (Fig. 5B) and discovered the 12 most influential “flag” molecules (top 12) for the UNICODE array to differentiate AD serum and HC serum (Table S2).

Traditionally, it has been challenging to use the top 12 as a single diagnostic marker. As each flag molecule carries a varying weight of significance and “echo” signatures present unique features for AD differentiation, smart analytics would be favorable to facilitate diagnostic performance. ML has emerged as a powerful tool for solving complex problems [44]. By using algorithms to train datasets and create self-learning models, ML has shown promise for diagnosing diseases with elusive etiology and diverse biomarkers [45]. To take full advantage of the UNICODE array, we employed ML to leverage the combined benefits of all top 12 compounds and to develop the best model for AD diagnosis. In this study, we used 31 AD and 31 HC samples and performed signature recording with the 12 “flag” molecules from the UNICODE array.

For ML, each compound can be considered an individual feature. Before we built the ML model using half-life information, we applied preprocessing steps to deal with the batch discrepancy caused by external factors [46,47]. After the data standardization, we performed principal component analysis to reduce the dimensions [48]. As shown in Fig. 5C and Fig. S5, AD and HC serums displayed different half-lifetime features in the top 12 array. To leverage the benefits of all 12 compounds, we performed ML with 7 models, namely, logistic regression, linear discriminant analysis (LDA), quadratic discriminant analysis (QDA), support vector machine (SVM), *K*-nearest neighbor (KNN), decision tree, and random forest (RF). We used *K*-fold cross-validation to train ML models, and basic metrics, including accuracy, precision, recall, F1, and area under the curve (AUC), were used for model evaluation. For each metric, we calculated the average value of 3 iterations. After tests with all ML models, we found that the LDA method provided fair separations between AD and HC (Fig. 5D), and we compared the outcomes of basic metrics to rank the top 5 models (logistic regression number of principal components [nPC] = 11, LDA nPC = 9, SVM nPC = 7, KNN nPC = 4, and RF nPC = 5). Among all models, RF showed the best performance with accuracy = 85.48%, precision = 85.00%, recall = 88.60%, F1 = 85.63%, and AUC = 90.24% (Fig. 5E and F and Fig. S6).

Furthermore, we explored whether a UNICODE array could differentiate AD from other diseases. We tested MS (*n* = 10), DB (*n* = 5) and rheumatoid arthritis (RA, *n* = 4) with the same protocol and analyzed their chemiluminescence decay half-lifetime features. Given the limited sample size, we prioritized LDA and QDA for their inherent ability to project high-dimensional data into 2-dimensional visualization spaces. While not optimized for classification performance, these methods allowed us to illustrate the potential of our approach for disease differentiation through interpretable low-dimensional representations. As shown in Fig. 5G and H and Figs. S7 and S8, a UNICODE array combined with classification algorithms could differentiate these diseases from AD. Similarly, we differentiated AD (*n* = 27) and the early stage of AD (*n* = 4, mild cognitive impairment due to AD [AD-MCI]) with a UNICODE array and classification algorithms (Fig. S9). Taken together, our data indicate that the UNICODE method has the potential for AD screening.

## Discussion

To the best of our knowledge, our UNICODE array methods are the first of their kind. Conventional methods are intended to identify biofluids’ signature proteins (or lipids/carbohydrates) through complicated and expensive technologies. However, physicochemical abnormality has been largely overlooked. Although fluorescence resonance energy transfer, surface plasmon resonance, and isothermal titration calorimetry are useful for detecting strong biomolecular interactions, these traditional approaches have a limited capacity to detect weak and/or cooperative interactions [36–38]. Also, these methods suffer from low throughput, limited sensitivity, and tediousness. Additionally, they generate only one readout per sample, failing to capture the diverse features and create disease signatures. Although we found that the fluorophore ANS could differentiate AD and HC serums, fluorescence imaging requires an external light source to excite and generate high background signals in most studies [21,23,49].

In our methods, instead of identifying the signatures, we use a compound array (UNICODE array) to reflect the signatures via signatures of chemiluminescence “echoes”. Compared to the conventional methods, the UNICODE array is straightforward. The samples (serum or other biofluids) require no additional processing steps, such as labeling or separating. This reduces the potential for operational errors. Importantly, our method is beneficial for preserving weak interactions, which might otherwise be disrupted by complicated sampling processes. These weak interactions could potentially be important features in specific diseases.

In this work, we selected AD as an example for proof-of-concept studies. AD is the most influential neurodegenerative disease that faces high demand for large-scale screening in an aging society [1–5]. Despite advances in high-cost positron emission computed tomography and single-molecule arrays for characterizing AD biomarkers, a simple, effective primary screening method is not at hand [50]. The UNICODE array provides a simple-to-handle and low-cost method for profiling AD signatures for further stratification, which could complement current diagnostic methods.

The transition of a groundbreaking idea normally takes decades to widespread clinical application. In the case of AD, the idea of blood diagnosis was reported decades ago and recently has been translated with the amyloid 40/42 test [51]. Our work represents the critical first step of the foundation of UNICODE for future advancements in AD diagnosis. However, our work still faces several challenges and further optimizations are needed: (a) ADLumin-1 exhibited a range of chemiluminescence features, including intensity, decay half-lifetime, and plateau (Fig. S3i). In this study, we focused on differentiating AD from HC by using one specific feature, the decay half-lifetime. We employed ML to improve the diagnosis performance via utilizing the half-lifetime of the decay of chemiluminescence signals. However, this approach has not fully taken advantage of our UNICODE arrays. Abundant information, such as the relationship between different signatures and the distribution of intensity, has not yet been used for ML due to limited sample sizes. Such information could be valuable for ML if a larger serum sample size is used. However, due to the small size of serum samples, our ML methods tend to overfit if all these metrics are input, which could compromise the diagnosis reliability of UNICODE arrays. With further optimization and a larger sample size, a specific interval of the signal decay profile or other metrics could be selected for faster diagnostic purposes and UNICODE arrays could enable comprehensive diagnosis evaluation with a tiny volume of biofluids. (b) In this study, we used only a top 12 array for differentiating AD and HC as a proof-of-concept study. It is conceivable that UNICODE arrays can be further improved via screening larger libraries to establish arrays of larger panels of compounds, such as 24-, 48-, and 96-compound panels. With larger and more diverse cohorts, we foresee that the UNICODE array could be a versatile platform to establish screening methods for other diseases through profiling certain signatures with specifically screened “flag” molecules and likely for different subtypes and stages. (c) In this study, we noticed that UNICODE could identify the interaction between albumin and its binders via screening of a small compound library and could be extended to other proteins, lipids, carbohydrates, and polynucleotides to screen binders. Further studies for exploring the drug screening capability of UNICODE would be highly desirable. Interestingly, we found that several compounds in the top 12 showed anti-AD effects and/or interacted with potential targets related to AD. For example, reportedly, compound 2 (glimepiride) has an anti-AD effect [16], and compounds 4 (etodolac) and 7 (ketoprofen) interact with the cyclooxygenase enzyme [49,50], which is considered a potential target for AD [51]. However, as AD is a complex disease with multiple contributing factors; further long-term fundamental studies are needed to address the important questions of which compounds and targets contribute to the UNICODE features of AD and whether these features could contribute to anti-AD drug discovery.

Despite these limitations and challenges, the UNICODE array provides a novel diagnosis principle and has great potential for clinical applications in the future.

In summary, we discovered the largely overlooked physicochemical abnormality of AD serum and developed a simple, versatile, and cost-effective method for screening AD. By using a “bat” probe and “flag” molecules to create chemiluminescence “echoes”, we enabled signature profiling of general serum components and subtle physicochemical environments. Furthermore, we screened the best “flag” molecules for depicting AD serum features and developed a UNICODE array for AD serums. By combining with ML, we successfully differentiated AD, HC, and other disease groups. Our study could provide new insights into biofluid abnormality and prototype tools for developing liquid biopsy diagnoses for AD and other diseases.

## Materials and Methods

### General information

All reagents were commercial products and used without further purification. ANS, human serum albumin (≥98%), IgG, and a BCG albumin assay kit were purchased from Sigma-Aldrich. A Pierce albumin serum depletion kit was purchased from Thermo Fisher. BDP, FA, and WAF were purchased from MedChemExpress. A compound library of approved drugs was purchased from TargetMol (L1000 approved drug library). Two types of anti-human-albumin antibodies were purchased from Thermo Fisher, which target amino acids 27 to 118 and amino acids 324 to 401 epitopes, respectively. A goat anti-rabbit IgG (H+L) horseradish peroxidase secondary antibody was purchased from Thermo Fisher. A nitrocellulose membrane (0.2 μm) was purchased from Bio-Rad. ADLumin-1 was synthesized and characterized by following our previously reported method. All experiments were approved by the Massachusetts General Hospital and carried out in accordance with the approved guidelines (protocol no. 2020P004007).

### ANS fluorescence spectrum measurement

A serum solution (100 μl) of a healthy or an AD single donor was diluted with PBS buffer (900 μl, 0.01 M, pH = 7.4). The diluted solution was titrated with different concentrations of ANS (20 to 80 μM), and the mixture was transferred into a cuvette. The fluorescence spectra (excitation = 350 nm, emission = 400 to 650 nm) were measured using an F-7100 fluorescence spectrometer (Hitachi, Japan).

### Albumin removal and LC–MS assay

In this assay, single-donor healthy or AD serum was subjected to BCG assays to quantify the albumin concentration. Briefly, 5 μl of diluted albumin standards, blank controls, and diluted serum samples were added into a 96-well clear-bottom plate. Next, 200 μl of BCG reagent from the assay kit was added to each well. The resulting mixture was incubated for 5 min at room temperature and subjected to absorbance measurement using a Molecular Devices SpectraMax microplate reader. The intensity at 620 nm was recorded, and the standard curves of different albumin concentrations were plotted. The albumin concentrations of the serum samples were calculated and normalized before albumin removal.

Albumin removal was conducted with a Pierce albumin serum depletion kit. Briefly, the albumin removal columns were prepared by applying resin slurry, rinsing with wash buffer, and centrifuging several times by following kit guidelines. Next, a single-donor healthy or AD serum solution (100 μl) was added into an albumin removal column, incubated for 2 min at room temperature, and centrifuged at 12,000 g for 1 min. The removal process was repeated twice to ensure maximal albumin binding. The preprocessed columns were placed into a 1.5-ml clear tube. Next, a cocktail solution (300 μl) of 50 μM BDP, FA, and WAF was loaded into the column, incubated for 2 min at room temperature, and centrifuged at 12,000 g for 1 min. This process was repeated twice to ensure maximal compound interaction with albumin. Finally, the flow-through solution was subjected to LC–MS analysis on an Agilent 1100 Series apparatus with an LC/mass selective detector trap and Daly conversion dynode detector. Standard samples of BDP, FA, and WAF were also subjected to LC–MS to confirm the standard retention peaks. The integral area of each peak was quantified and normalized with the integral area of the FA peak.

### Dot blot assay of epitope mapping

A solution of single-donor healthy or AD serum (12 μl) was diluted with PBS buffer (588 μl). A nitrocellulose membrane (0.2 μm) was cut and soaked in PBS buffer (0.01 M, pH = 7.4) for 30 min, followed by applying it on a 96-well Bio-Dot Microfiltration Apparatus. A diluted serum solution (90 μl) was immediately added into each well and immobilized on the nitrocellulose membrane under vacuum. The membrane was blocked by 5% nonfat milk for 1 h, followed by incubation with an anti-albumin antibody (1:2,000 diluted with 5% nonfat milk) at 4 °C overnight. The membrane was washed with Tris-buffered saline with 0.1% Tween (TBS-T) solution for 3 × 10 min and then incubated with horseradish peroxidase-conjugated secondary antibody (1:2,000 diluted with 5% nonfat milk) for 1.5 h at room temperature. After washing with TBS-T for 3 × 10 min, the membrane was incubated with a Pierce ECL western blotting substrate for 1 min and observed by an IVIS Spectrum imaging system (PerkinElmer) with a blocked excitation filter and an opened emission filter. A region of interest was drawn around each dot, and the chemiluminescence intensity was quantified.

### Chemiluminescence signal profiling and UNICODE assay

Each major serum component, including albumin, IgG, fibrinogen, or hemoglobin, was diluted with PBS buffer to give a final concentration of 10 μM. For mimic serum studies, a mixture of albumin (600 μM) and IgG (67 μM) was prepared to mimic serum conditions and diluted 50-fold with PBS buffer for further investigation.

A diluted solution (50 μl) was added into each well of a black 96-well plate, followed by adding a solution (2.5 μl) of a compound (final concentration = 2 μM) from the approved drug library (TargetMol, L1000 approved drug library). The plate was sealed and gently shaken for 30 min, followed by adding an ADLumin-1 solution (2.5 μl, final concentration = 25 μM). The plate was gently shaken for 1 min and immediately imaged with the IVIS Spectrum imaging system (PerkinElmer) from 5 to 180 min with a blocked excitation filter and an opened emission filter. The chemiluminescence intensity of each well was quantified under grid region of interest mode. The decay profile was analyzed with GraphPad Prism, and the half-lifetime was quantified under a nonlinear regression phase decay mode.

For the UNICODE assay, a solution (30 μl) of single-donor healthy or AD serum was diluted with PBS buffer (1,470 μl). A diluted solution (50 μl) was added into each well of a black 96-well plate, followed by adding a compound solution (2.5 μl, final concentration = 2 μM). The plate was sealed and gently shaken for 30 min and treated with ADLumin-1 by following the above imaging procedures.

### ML analysis

Preprocessing steps were conducted to deal with batch information and avoid overfitting. The following assumption on half-life was applied:

$$x_{\mathrm{ij}}=\mu_{i}+\Delta_{s}+\varepsilon_{\mathrm{ij}}$$ (1)

Here, $x_{\mathrm{ij}}$ stands for the half-life of the jth serum from batch i. $\mu_{i}$ is the baseline value assigned to each batch to account for differences in environment and equipment that may introduce measurement errors. This variable is mainly intended to characterize the external factors in the batch of experiments. In this model, we assume that external factors have a linear effect on the results. $\Delta_{s}$ $\left(\Delta_{0}=\Delta_{\mathrm{HC}},\Delta_{1}=\Delta_{\mathrm{AD}}\right)$ is the overall half-life difference of HC and AD. $\varepsilon_{\mathrm{ij}}$ stands for the error consisting of experimental error and individual difference, which is independent and normally distributed with a mean of zero. A straightforward way was applied to define a new variable $z_{\mathrm{ij}}≔x_{\mathrm{ij}}-\mu_{i}=\Delta_{s}+\varepsilon_{\mathrm{ij}}$. If we reindex these serums by flattening them, the formula becomes $z_{m}=\Delta_{s}+\varepsilon_{m}$, where m stands for the mth serum from all serums. Now the objective is to calculate $\mu_{i}$:

$$\mu_{i}=x_{\mathrm{ij}}-\Delta_{s}-\varepsilon_{\mathrm{ij}}$$ (2)

where $\Delta_{s}$ is unknown. Without loss of generality, we assume $\Delta_{0}+\Delta_{1}=0$. Furthermore, the sets of indices of HC serums and AD serums are denoted by $A_{0}$ and $A_{1}$ separately. Then, we take the average of Eq. 2 over $A_{0}$ and $A_{1}$ separately to get

$$\mu_{i}=\frac{1}{\left|A_{0}\right|}\sum_{j\in A_{0}}x_{\mathrm{ij}}-\Delta_{0}-\frac{1}{\left|A_{0}\right|}\sum_{j\in A_{0}}\varepsilon_{\mathrm{ij}}$$ (3)

$$\mu_{i}=\frac{1}{\left|A_{1}\right|}\sum_{j\in A_{1}}x_{\mathrm{ij}}-\Delta_{1}-\frac{1}{\left|A_{1}\right|}\sum_{j\in A_{1}}\varepsilon_{\mathrm{ij}}$$ (4)

Then, we take the average of Eqs. 3 and 4 and apply the expected value.

$$\mu_{i}=E\left(\frac{1}{2}\left(\frac{1}{\left|A_{0}\right|}\sum_{j\in A_{0}}x_{\mathrm{ij}}+\frac{1}{\left|A_{1}\right|}\sum_{j\in A_{1}}x_{\mathrm{ij}}\right)\right)$$ (5)

Here, the terms $\Delta_{0}$ and $\Delta_{1}$ are eliminated because $\Delta_{0}+\Delta_{1}=0$. The terms $\varepsilon_{\mathrm{ij}}$ are eliminated because their expected values are 0. Hence, the approximated $\mu_{i}$ can be calculated as

$$\begin{array}{c} \begin{array}{c} \begin{array}{c} \frac{1}{2}\left(\text{average of}\;x_{\mathrm{ij}}\;\text{among}\;\mathrm{all}\;\mathrm{HC}\;\text{serums}+\text{average of}\;x_{\mathrm{ij}}\;\text{among}\;\mathrm{all}\;\mathrm{AD}\;\text{serums}\right) \end{array} \end{array} \end{array}$$ (6)

Principal component analysis was used to reduce dimension reduction. To train the model, *K*-fold cross-validation was chosen to train the model. More specifically, for each ML algorithm in the following sections, the dataset was shuffled randomly and split into 3 groups. Each unique group was taken as the test set and the remaining group was taken as the training set. A model was fit on the training set and evaluated on the test set. Finally, the average of the metrics in 3 iterations will show the overall performance of the certain algorithm. Various methods including logistic regression, LDA, QDA, SVM, KNN, decision tree, and RF were used for model training and metrics including accuracy, precision, recall, F1, and AUC were used to compare model performance.

### Statistical analysis

Quantitative data shown as mean ± standard error of the mean were analyzed by the GraphPad Prism 8.0 software. *P* values were determined by unpaired 2-tailed Student *t* tests. The differences were considered significant when *P* ≤ 0.05.

## Data Availability

All data are available in the main text or the Supplementary Materials.
