# Supplementary material for: Creating Chemiluminescence Signature Arrays Coupled with Machine Learning for Alzheimer’s Disease Serum Diagnosis
Source: Research (Wash D C). 2025 May 12;8:0653. doi: 10.34133/research.0653 (PMC12067928; doi:10.34133/research.0653)
Supplement: Supplementary 1 — Figs. S1 to S9 Tables S1 and S2 [file research.0653.f1.zip › SI Fig. QDA MS DB RA.pdf]

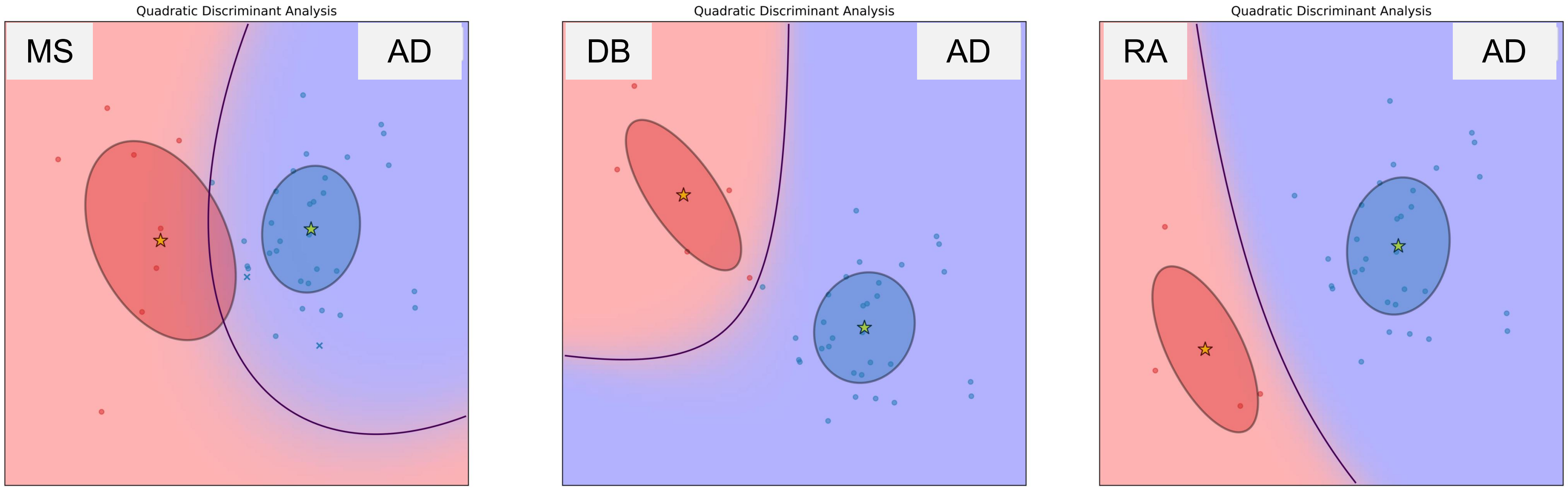

Quadratic Discriminant Analysis
MS
AD
Quadratic Discriminant Analysis
DB
AD
Quadratic Discriminant Analysis
RA
AD
