# Supplementary material for: Creating Chemiluminescence Signature Arrays Coupled with Machine Learning for Alzheimer’s Disease Serum Diagnosis
Source: Research (Wash D C). 2025 May 12;8:0653. doi: 10.34133/research.0653 (PMC12067928; doi:10.34133/research.0653)
Supplement: Supplementary 1 — Figs. S1 to S9 Tables S1 and S2 [file research.0653.f1.zip › SI Table1.pdf]

1	2	3	4	5	6	7	8	9	10	11	12
a	122892-31-3	1446502-11-9	313-67-7	119-36-8	120-51-4	127-47-9	130209-82-4	141-94-6	151-67-7	2078-54-8	a
	Itopride hydrochloride	Enasidenib	Aristolochic Acid	Methyl salicylate	Benzyl benzoate	Retinyl acetate	Latanoprost	Hexetidine	Anestan	Propofol	
b	30544-47-9	4180-23-8	483-63-6	51-03-6	543-82-8	555-57-7	583-03-9	58-95-7	59-02-9	637-07-0	b
	Etofenamate	Trans-Anethole	Crotamiton	Piperonyl butoxide	2-Amino-6-methylheptane	Pargyline	Fenipentol	Vitamin E Acetate	Vitamin E	Clofibrate	
c	79-55-0	84-80-0	97-53-0	132-60-5	72956-09-3	81-23-2	120511-73-1	98717-15-8	985-13-7	57-66-9	c
	Pempidine	Vitamin K1	Eugenol	Cinchophen	Carvedilol	Dehydrocholic acid	Anastrozole	Ropivacaine hydrochloride	Ethaverine hydrochloride	Probenecid	
d	51146-56-6	5560-59-8	132203-70-4	2030-63-9	132-20-7	53-86-1	5987-82-6	38194-50-2	54910-89-3	637-58-1	d
	Dexibuprofen	Alverine citrate	Cilnidipine	Clofazimine	Pheniramine maleate	Indometacin	Oxybuprocaine hydrochloride	Sulindac	Fluoxetine	Pramoxine hydrochloride	
e	58186-27-9	121-54-0	90357-06-5	183321-74-6	78213-16-8	6398-98-7	68767-14-6	88495-63-0	964-52-3	85-79-0	e
	Idebenone	Benzethonium chloride	Bicalutamide	Erlotinib	Diclofenac diethylamine	Amodiaquine hydrochloride	Loxoprofen	Artesunate	Moxisylyte hydrochloride	Dibucaine	
f	27220-47-9	2295-58-1	130-61-0	73963-72-1	60-87-7	57-96-5	169590-42-5	5786-21-0	550-99-2	84-22-0	f
	Econazole	Flopropione	Thioridazine hydrochloride	Cilostazol	Promethazine	Sulfinpyrazone	Celecoxib	Clozapine	Naphazoline hydrochloride	Tetryzoline	
g	50-50-0	31430-15-6	630-93-3	126-27-2	50-42-0	1218-35-5	152-43-2	129938-20-1	471-53-4	84-17-3	g
	Estradiol benzoate	Flubendazole	Phenytoin sodium	Oxethazaine	Adiphenine hydrochloride	Xylometazoline hydrochloride	Quinestrol	Dapoxetine HCl	Enoxolone	Dienestrol	
h	50-04-4	58066-85-6	138982-67-9	52-86-8	18010-40-7	89778-26-7	97-77-8	3820-67-5	22881-35-2	132-18-3	h
	Cortisone acetate	Miltefosine	Ziprasidone hydrochloride monohydrate	Haloperidol	Bupivacaine hydrochloride	Toremifene	Disulfiram	Glafenine	Famprofazone	Diphenylpyraline hydrochloride	
