# Supplementary material for: Creating Chemiluminescence Signature Arrays Coupled with Machine Learning for Alzheimer’s Disease Serum Diagnosis
Source: Research (Wash D C). 2025 May 12;8:0653. doi: 10.34133/research.0653 (PMC12067928; doi:10.34133/research.0653)
Supplement: Supplementary 1 — Figs. S1 to S9 Tables S1 and S2 [file research.0653.f1.zip › SI Table2.pdf]

Flag No.	Compound Name
1	Clofazimine
2	Glimepiride
3	Chloroambucil
4	Etodolac
5	Anetholetrithione
6	Carbamazepine
7	(S)-(+)-Ketoprofen
8	MenadiolDiacetate
9	AbirateroneAcetate
10	Tideglusib
11	Entospletinib
12	DinoprostTromethamine
