# Supplementary material for: Creating Chemiluminescence Signature Arrays Coupled with Machine Learning for Alzheimer’s Disease Serum Diagnosis
Source: Research (Wash D C). 2025 May 12;8:0653. doi: 10.34133/research.0653 (PMC12067928; doi:10.34133/research.0653)
Supplement: Supplementary 1 — Figs. S1 to S9 Tables S1 and S2 [file research.0653.f1.zip › Supplementary Information.docx]

**Figure S1. a)** Representative ANS titration assay result for HC and AD serum (n = 3). Different concentrations of ANS dye (20-80 μM) were added into the serum dilution samples and the fluorescence intensity were recorded. This result suggested that the total surface hydrophobicity of AD serum was different from HC serum, and the AD serum had less accessible surface hydrophobicity. b,c) Representative fluorescence emission spectra of ANS with multiple sclerosis (MS) (b) or diabetes (DB) (c) and HC serums (n = 3). These serums also showed decreased ANS intensity compared to HC but no apparent wavelength shift, suggesting that both similarities and differences in physicochemical abnormalities could exist in different diseases.

**Figure S2.** Representative TEM images of IgG under heating conditions. The conformation of IgG secondary structure showed perturbation at 25 ℃and 60 ℃, and structure destruction at 80℃.

**Figure S3**. a,b) Chemiluminescence decay profile of different pH conditions ranging from 5.5 to 8 without (a) or with (b) human serums detected by ADLumin-1. c,d) Chemiluminescence decay profile of different viscosity adjusted by 0-80% (v/v) of glycine/PBS without (c) or with (d) human serums detected by ADLumin-1. e,f) Chemiluminescence decay profile of different concentrations of hydrogen peroxide (0-100 μM) without (e) or with (f) human serums detected by ADLumin-1. g) Chemiluminescence decay profile of ADLumin-1 at different temperatures (25-80 ℃). h,i ) Chemiluminescence signal intensity (h) and decay features (i) of AD and HC serums (n = 14 for AD and n = 16 for HC) with ADLumin-1. Compared to HC, the serums of AD showed lower chemiluminescence intensities and shorter half-life times.

**Figure S4.** a) “Flag” molecule screening experiments of representative compounds of “non-flag” molecules (No.2, 14, 26, 84) and “flag” molecule (No.38) in three replicates. b) Chemiluminescence decay half-life time of albumin and representative “non-flag” molecules (No.2, 14, 26, 84) and “flag” molecule (No.38) detected by different concentrations of ADLumin-1 (12.5-50 μM). c-f) Chemiluminescence “echo” signatures of various serum components. Chemiluminescence decay half-life time of fibrinogen, alpha-1-acid glycoprotein (AGP), hemoglobin, and transferrin were measured with ADLumin-1 and 96-well array. Each serum component has unique echo signatures to reflect its features.

**Figure S5.** Chemiluminescence decay half-life time differences of serum samples from HC and AD by using UNICODE top-12 array.

**Figure S6**. Comprehensive metrics results of machine learning models. Principal Component Analysis (PCA) was first used to reduce dimension reduction. Top-12 UNICODE data for AD and HC serum were trained with various machine learning methods, including logistic regression, linear discriminant analysis (LDA), quadratic discriminant analysis (QDA), support vector machine (SVM), K-nearest neighbor (KNN), decision tree, and random forest. Metrics including accuracy, precision, recall, f1, AUC were used to compare the model performance. Each bar is the average of the metrics in 3 iterations.

**Figure S7.** Chemiluminescence decay half-life time differences of serum samples from AD (n = 31) and MS (n = 10), DB (n = 5), RA (n = 4) serums by using UNICODE top-12 array.

**Figure S8**. Classification of AD (n = 31) and MS (n = 10), DB (n = 5), RA (n = 4) serums signatures with QDA method.

**Figure S9.** Classification of AD (n = 27) and AD-MCI (n = 4) disease serums signatures with LDA or QDA method.

**Table S1.** Compound information for the 96-well array.

**Table S2.** List of the top-12 flag molecules for AD serum.
