# Supplementary figures and images for: Creating Chemiluminescence Signature Arrays Coupled with Machine Learning for Alzheimer’s Disease Serum Diagnosis
Source: Research (Wash D C). 2025 May 12;8:0653. doi: 10.34133/research.0653 (PMC12067928; doi:10.34133/research.0653)

Linear Discriminant Analysis

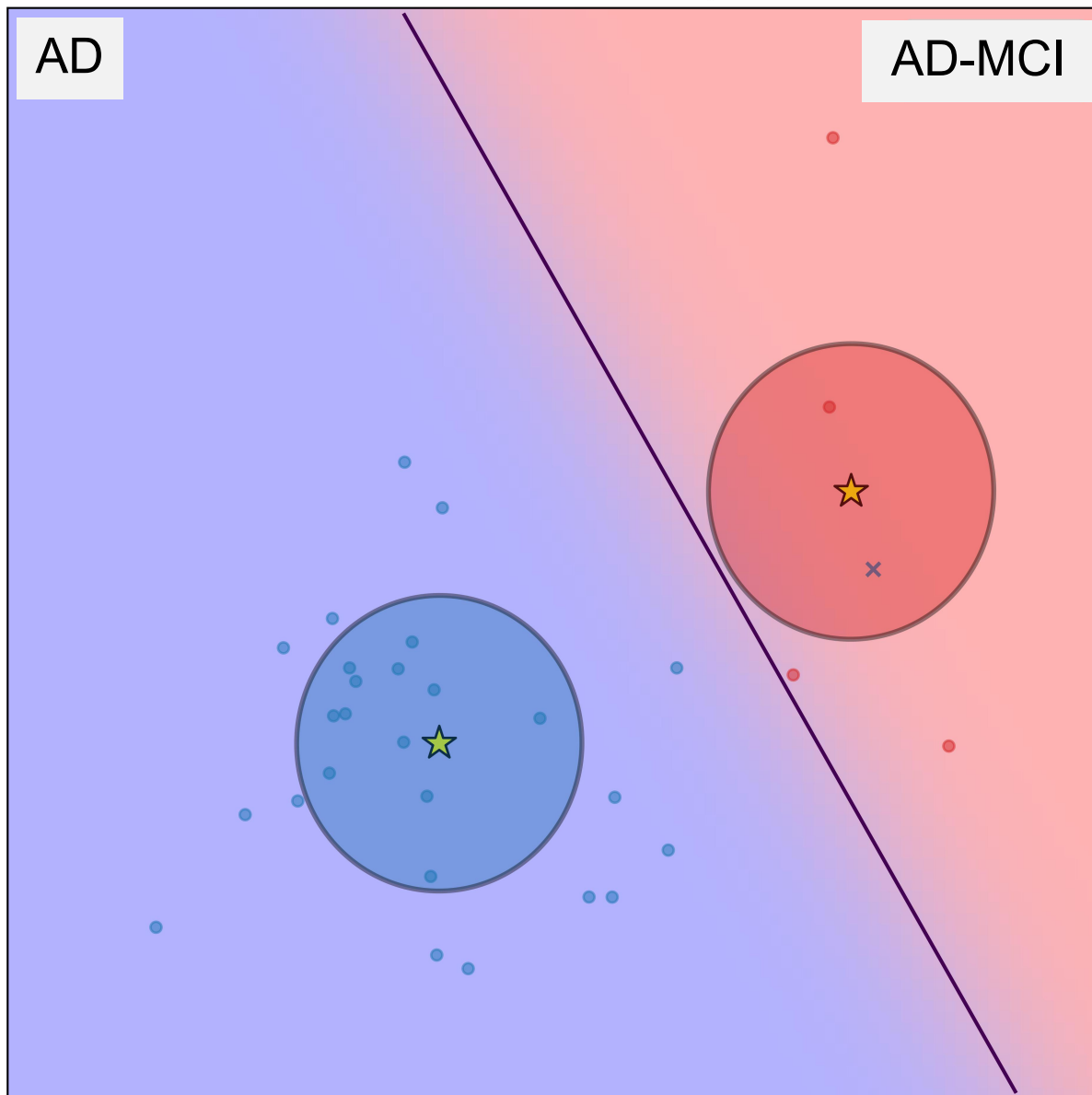

Quadratic Discriminant Analysis

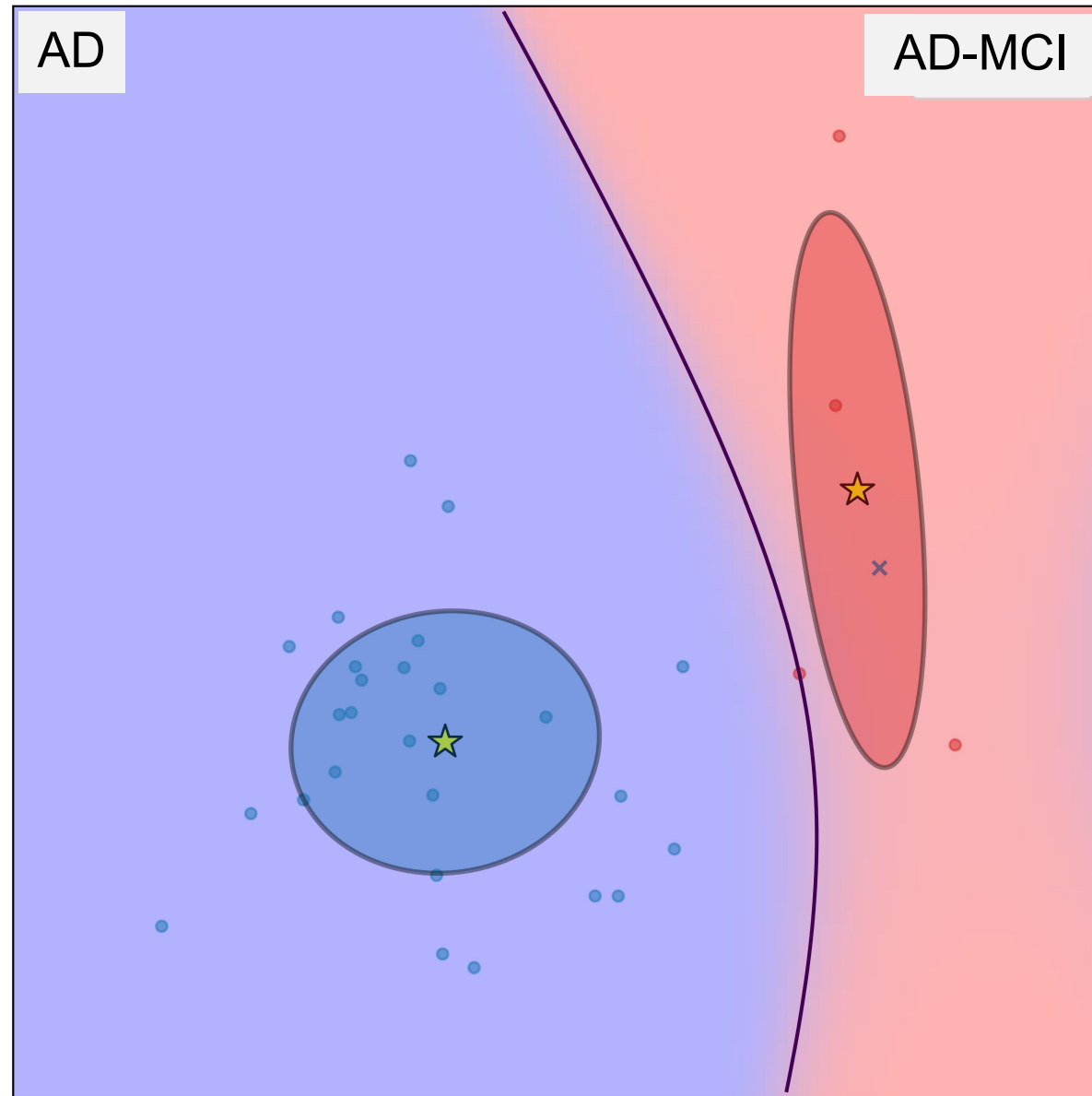

Supplement: Supplementary 1 — Figs. S1 to S9 Tables S1 and S2 [file research.0653.f1.zip › Fig SI AD MCI-AD.pdf]

**a**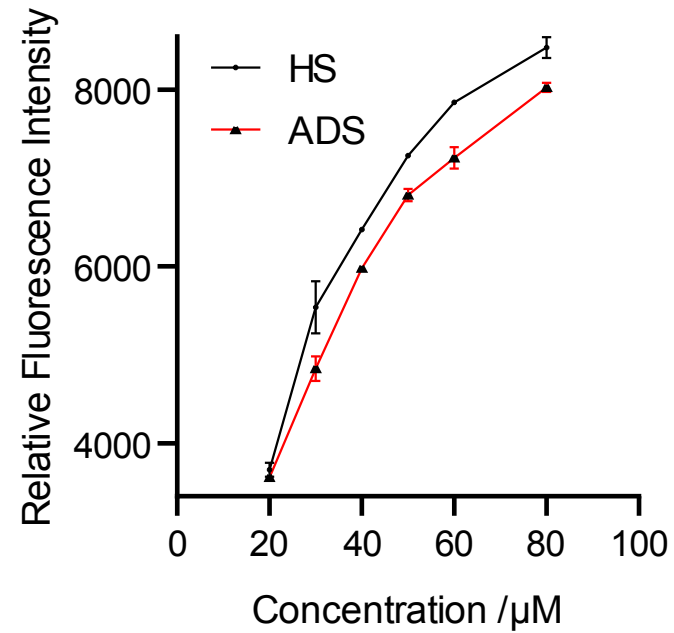**b**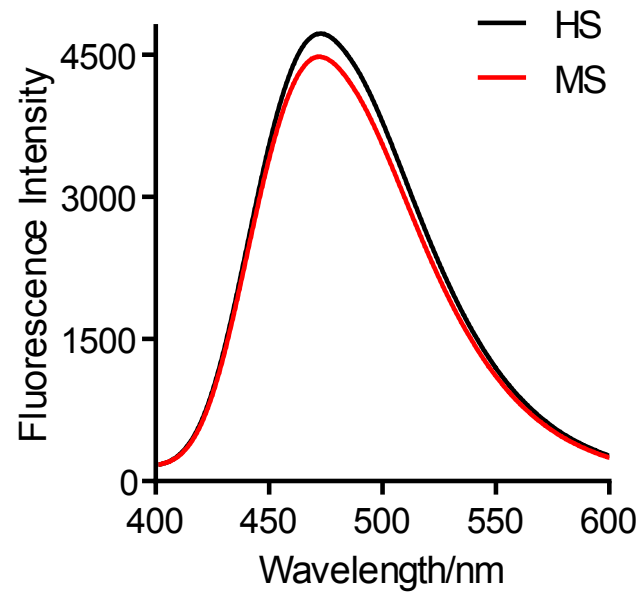**c**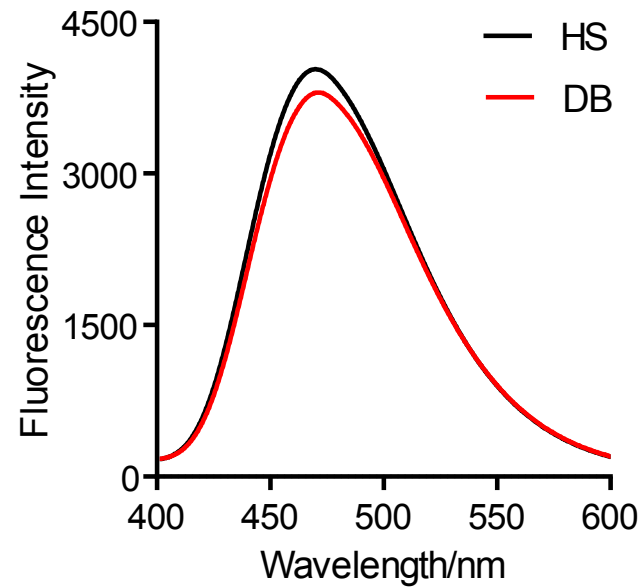

Supplement: Supplementary 1 — Figs. S1 to S9 Tables S1 and S2 [file research.0653.f1.zip › SI Fig 1.pdf]

IgG 25 °C

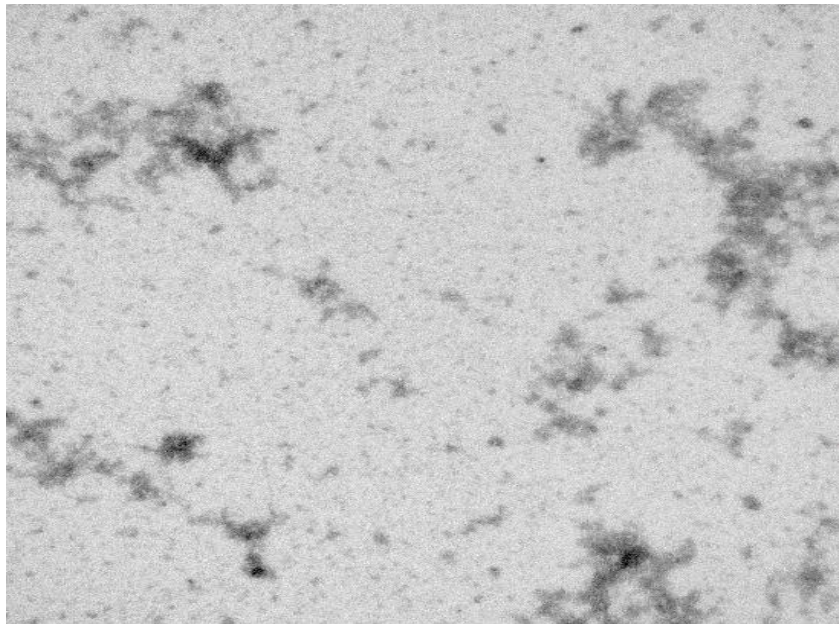

IgG 60 °C

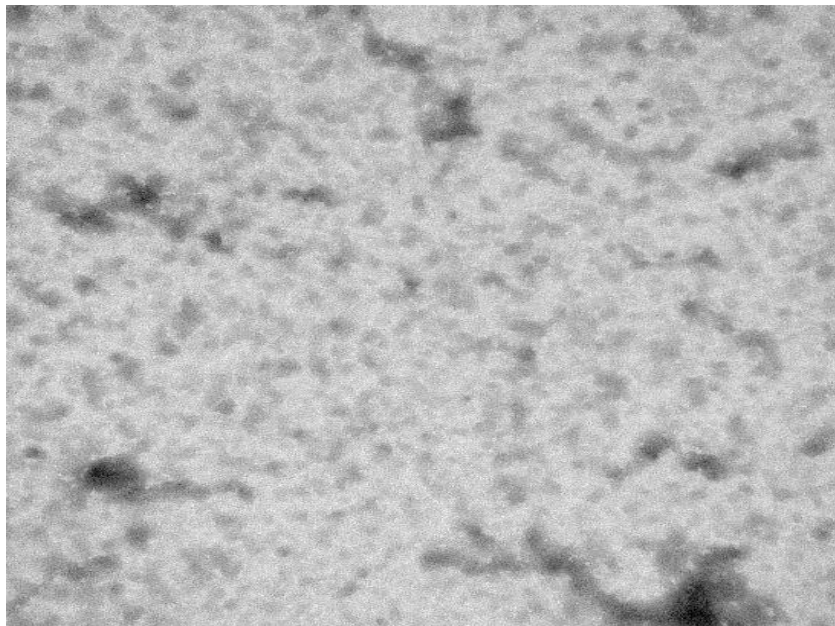

IgG 80 °C

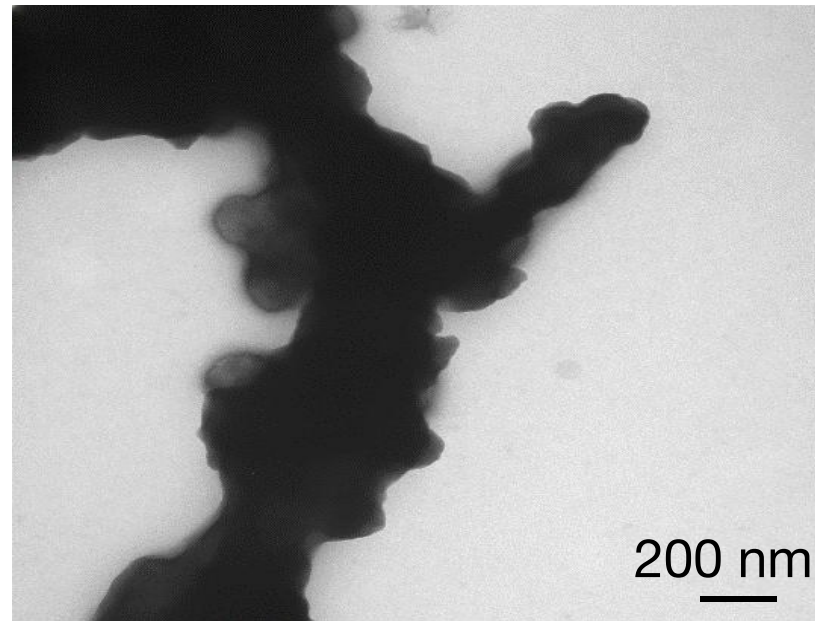

Supplement: Supplementary 1 — Figs. S1 to S9 Tables S1 and S2 [file research.0653.f1.zip › SI Fig 2.pdf]

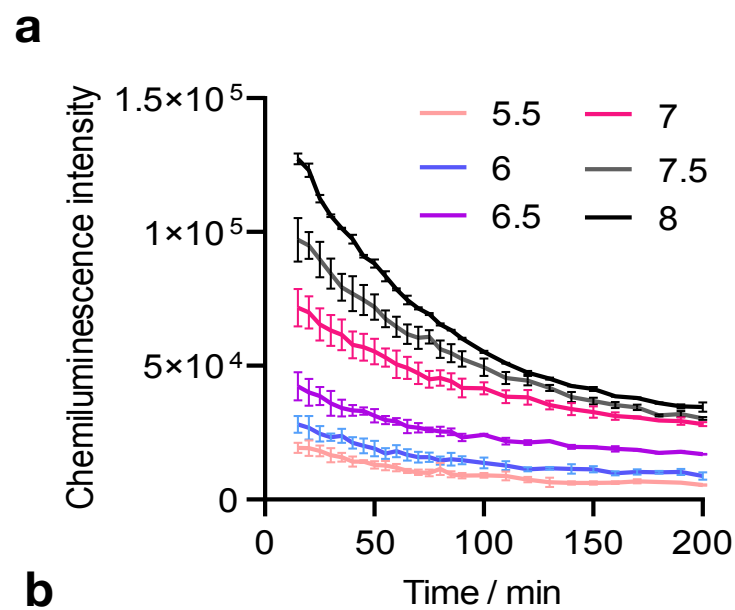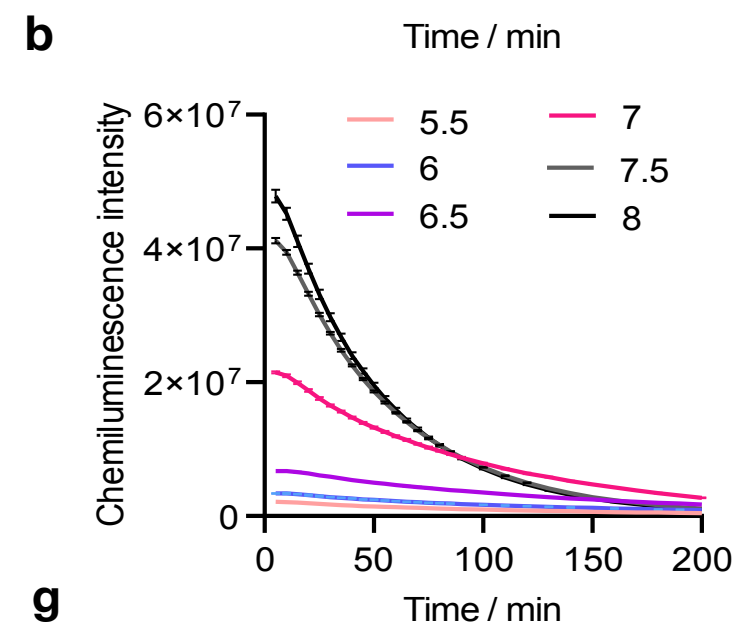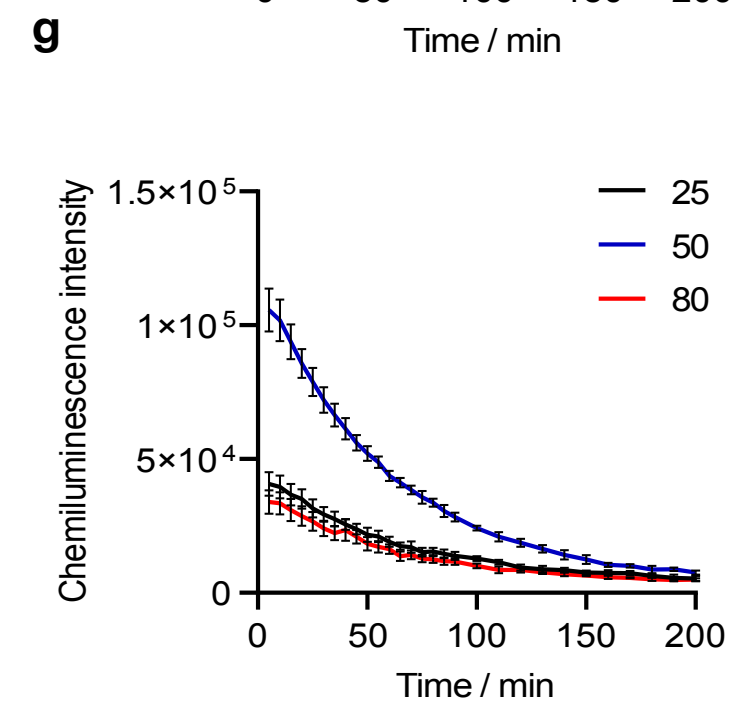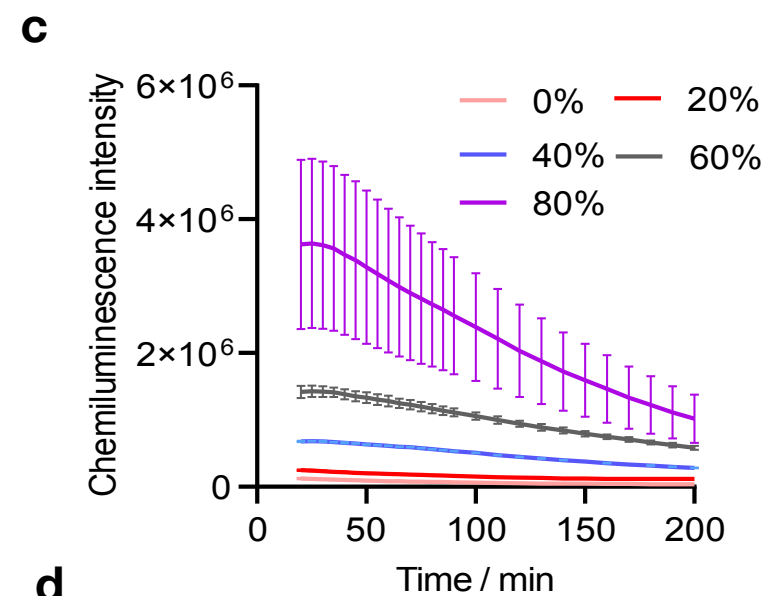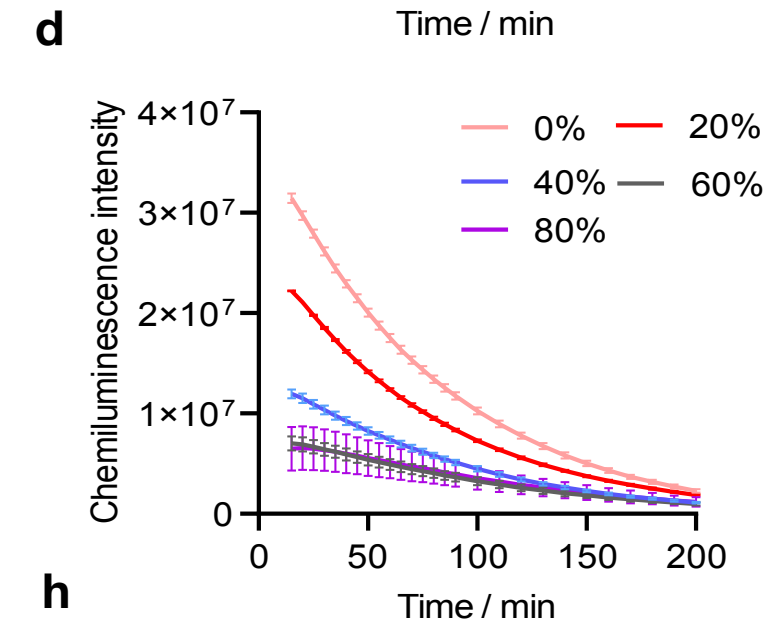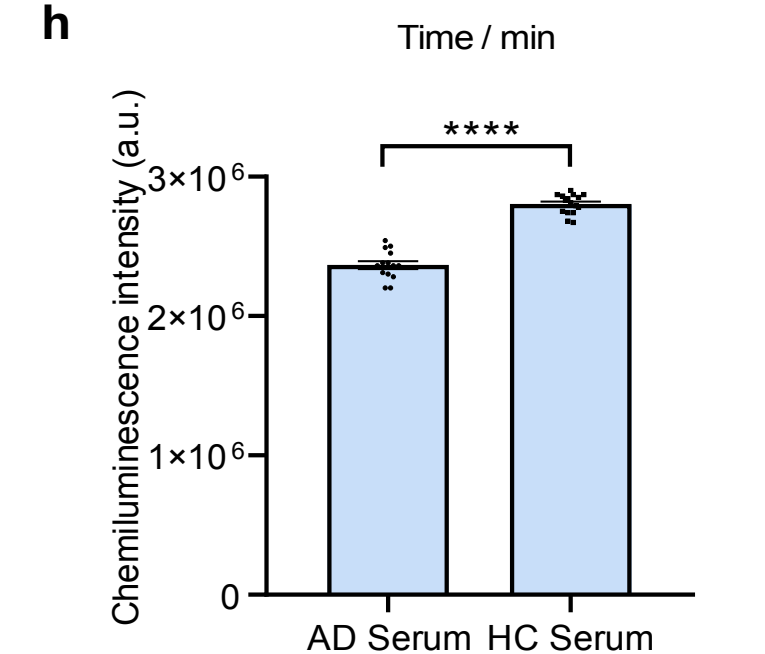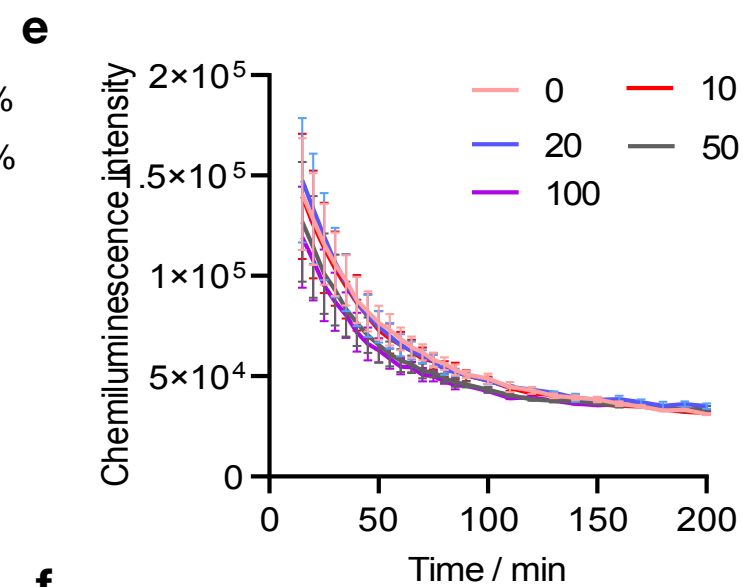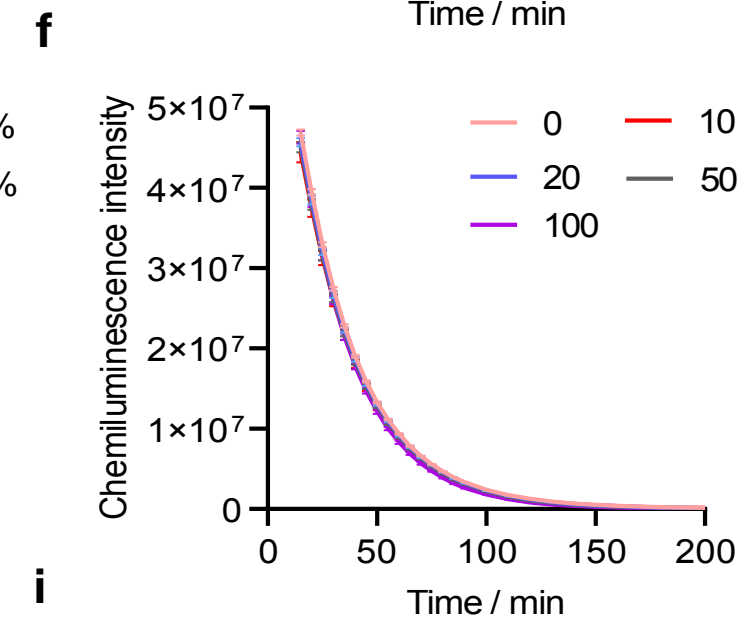

| metrics   | AD      | HC      |
|-----------|---------|---------|
| Y0        | 2393502 | 2914371 |
| Plateau   | 102642  | 73099   |
| K         | 0.02277 | 0.01454 |
| Half Life | 30.44   | 47.67   |
| Tau       | 43.92   | 68.77   |
| Span      | 2290859 | 2841272 |
| R squared | 0.9917  | 0.9895  |

Supplement: Supplementary 1 — Figs. S1 to S9 Tables S1 and S2 [file research.0653.f1.zip › SI Fig 3.pdf]

**a**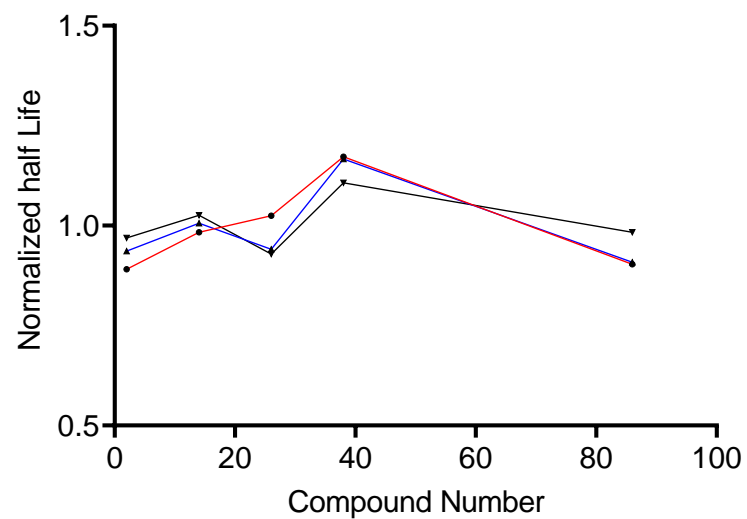**b**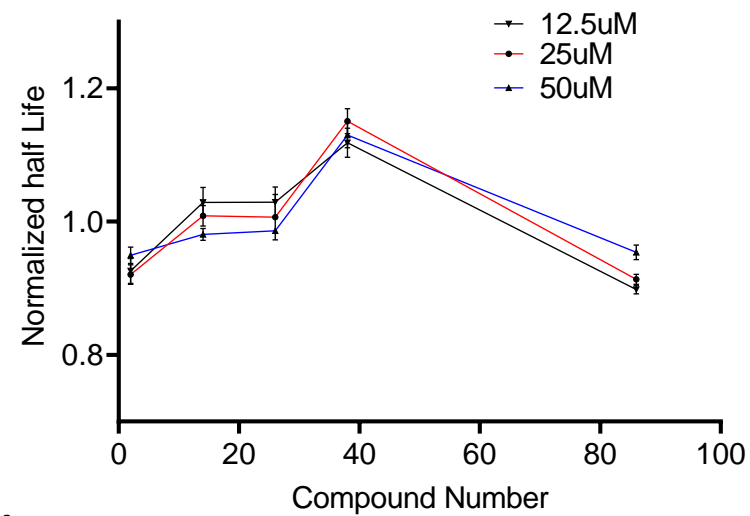**c**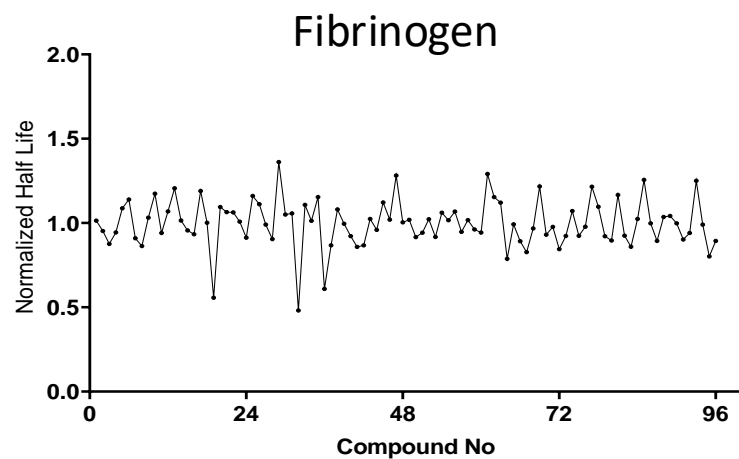**d**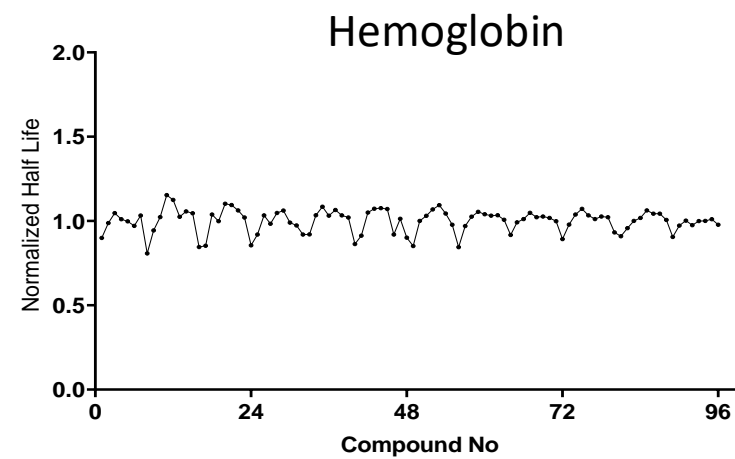**e**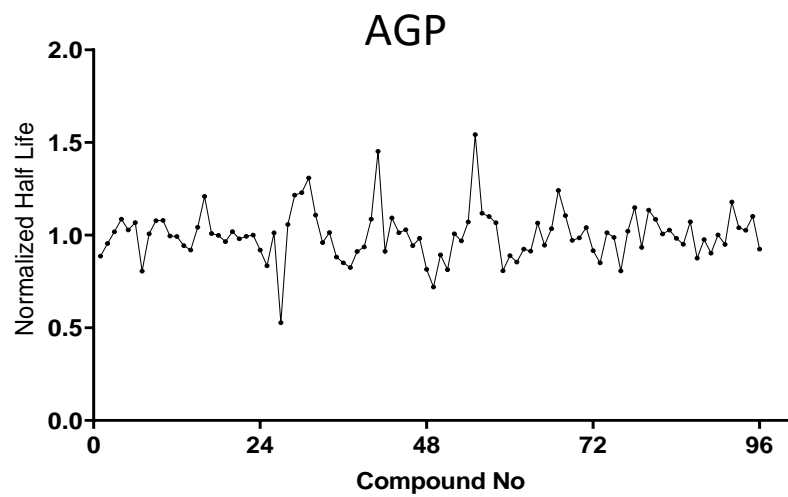**f**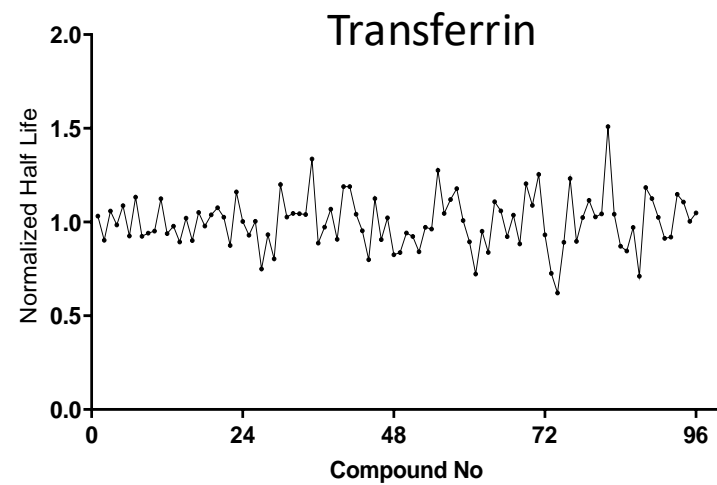

Supplement: Supplementary 1 — Figs. S1 to S9 Tables S1 and S2 [file research.0653.f1.zip › SI Fig 4.pdf]

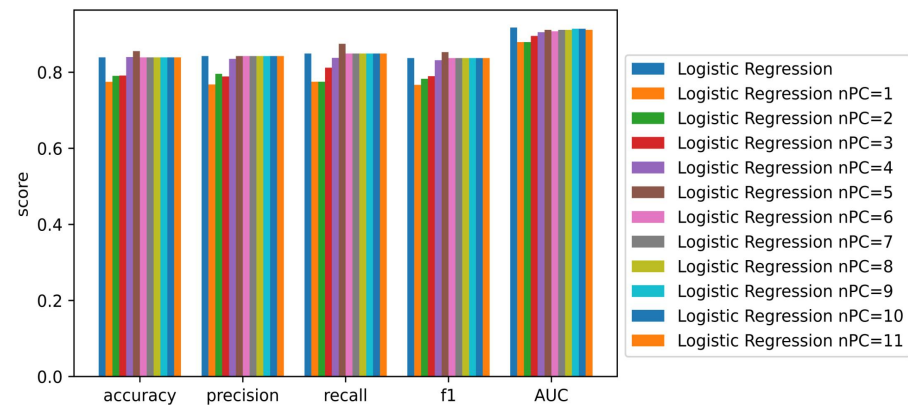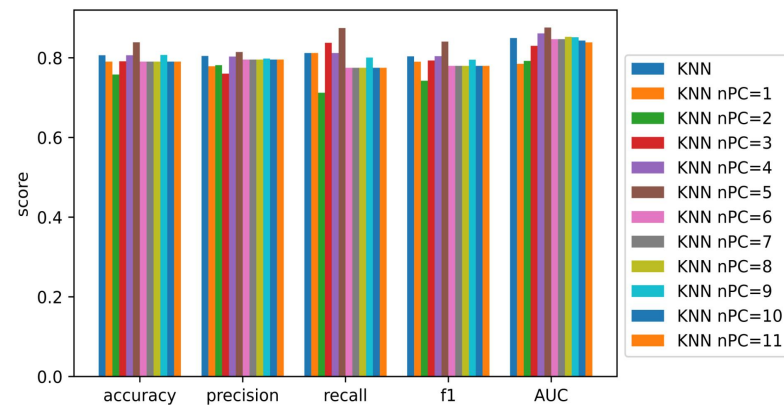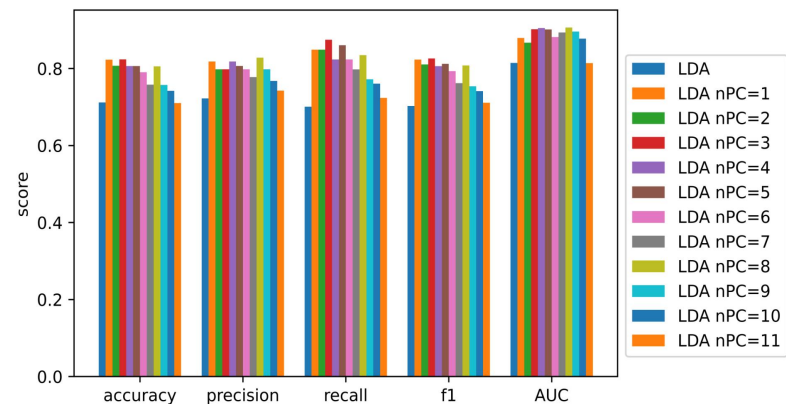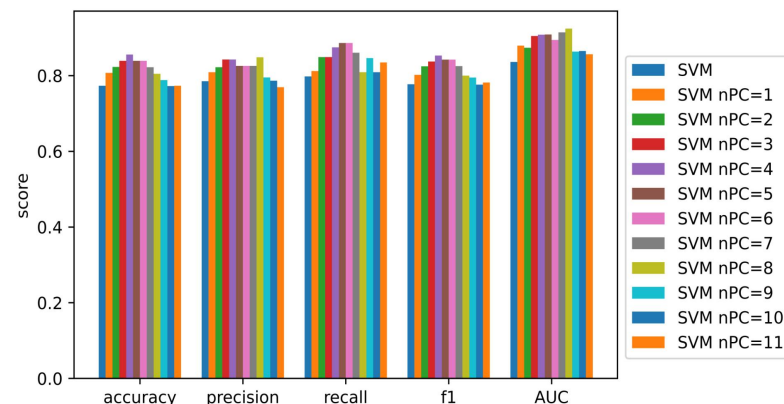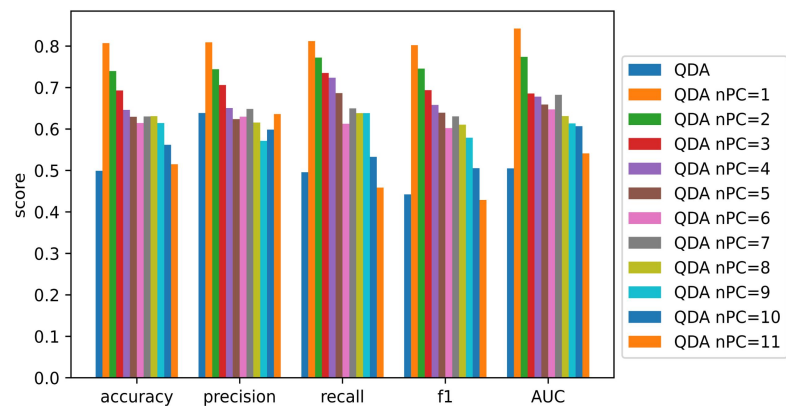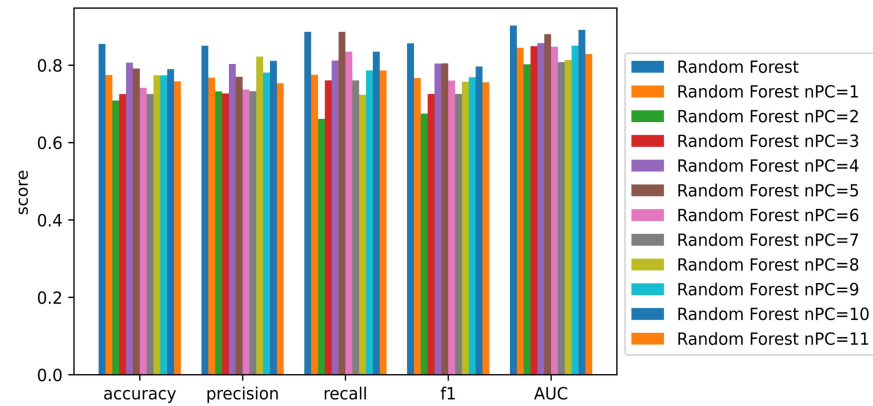

Supplement: Supplementary 1 — Figs. S1 to S9 Tables S1 and S2 [file research.0653.f1.zip › SI Fig 6.pdf]

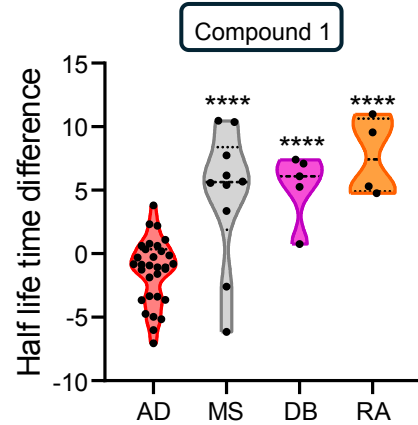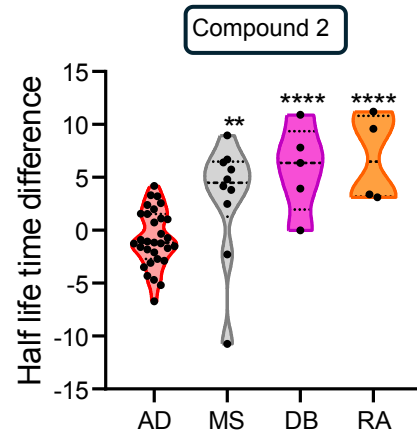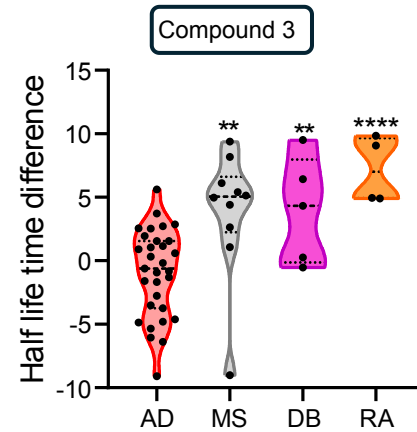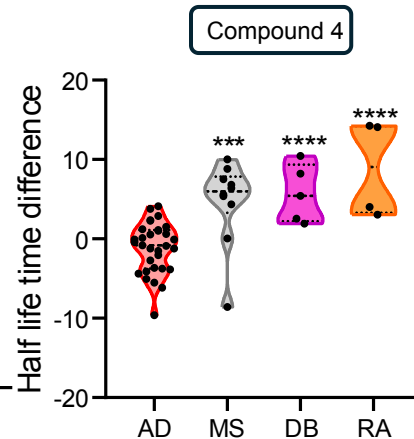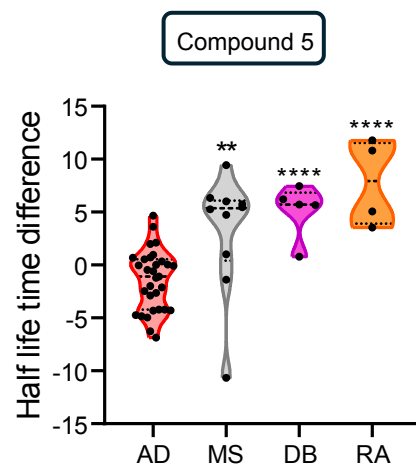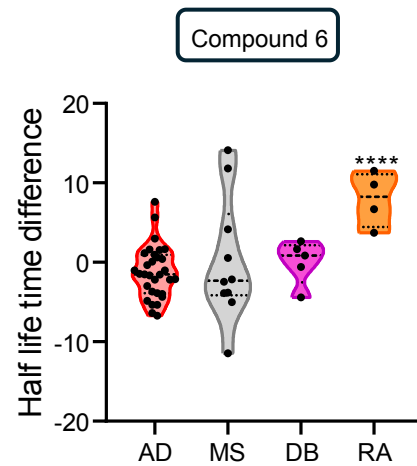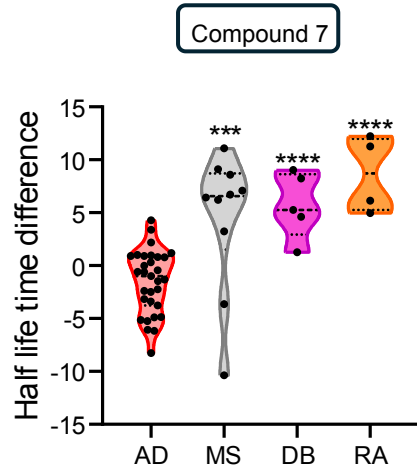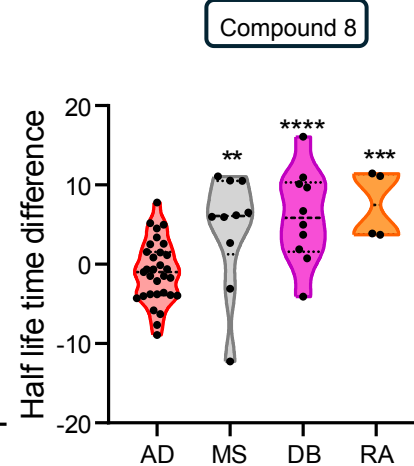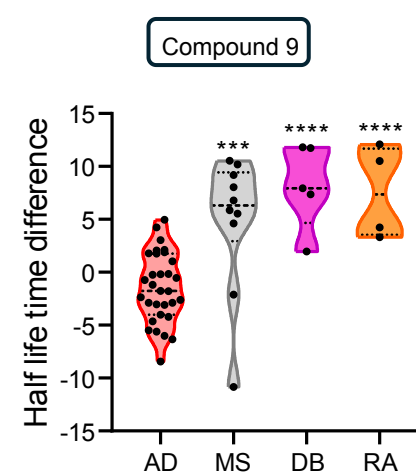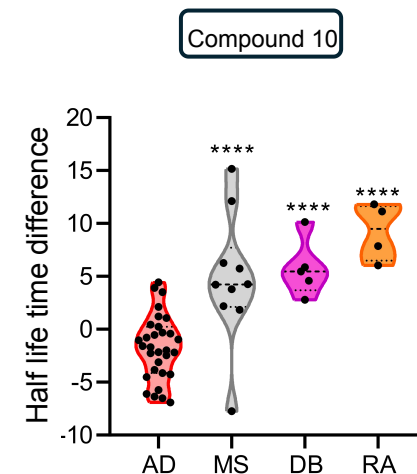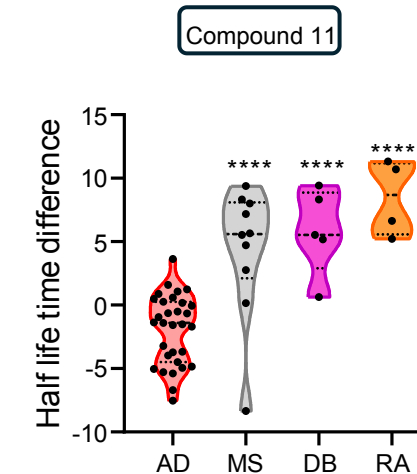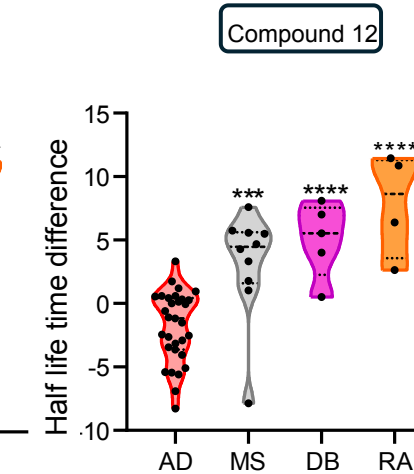

Supplement: Supplementary 1 — Figs. S1 to S9 Tables S1 and S2 [file research.0653.f1.zip › SI Fig. AD MS DB RA top12.pdf]

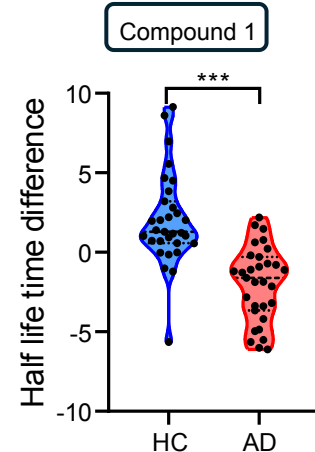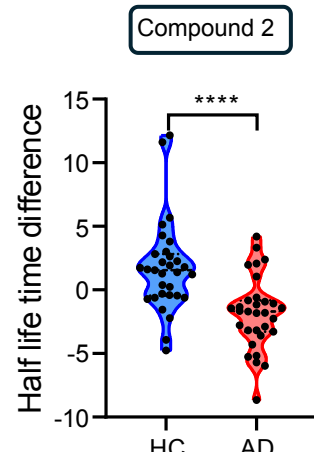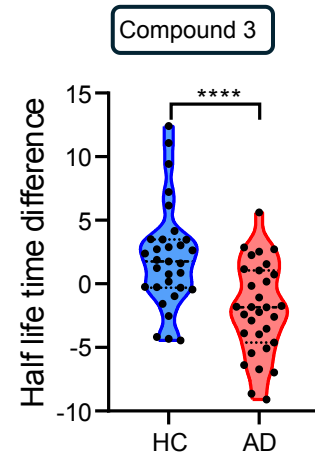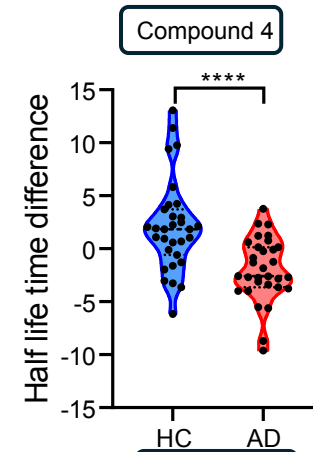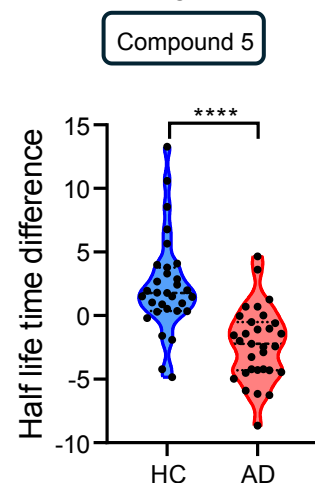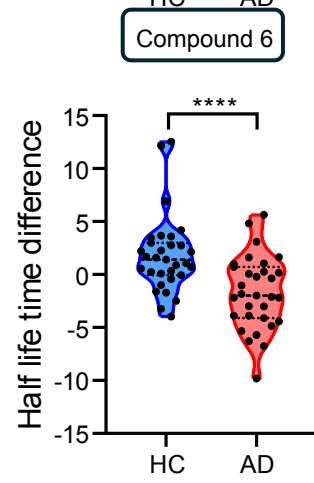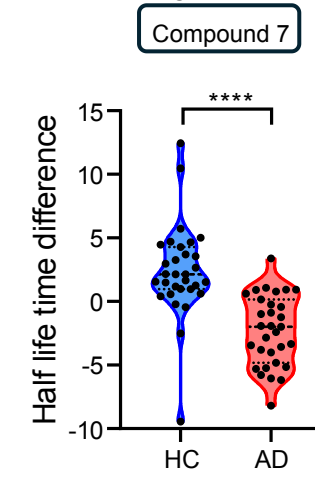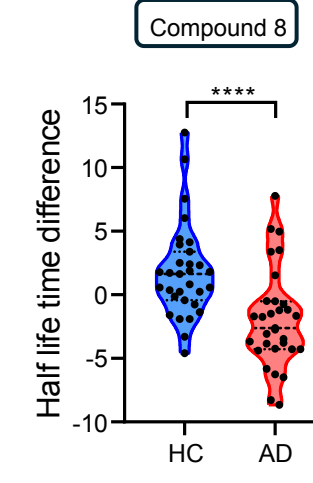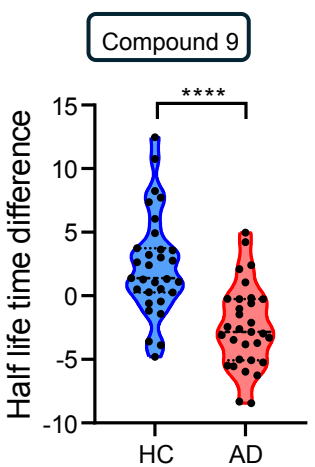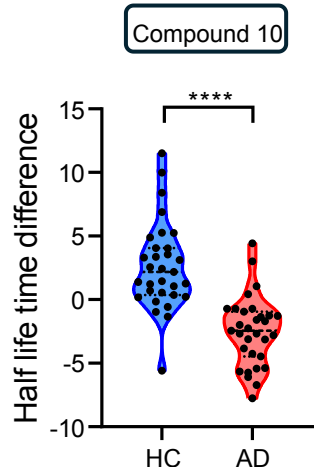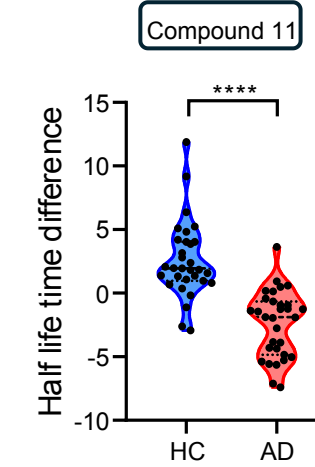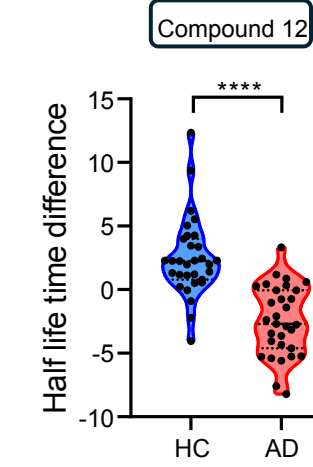

Supplement: Supplementary 1 — Figs. S1 to S9 Tables S1 and S2 [file research.0653.f1.zip › SI Fig. HC top12.pdf]
